# N‐Heterocyclic Carbenes on a III‐V Semiconductor: From Chain Formation to Ordered Monolayers

**DOI:** 10.1002/anie.202511094

**Published:** 2025-09-22

**Authors:** Martin Franz, Ankita Das, Sandhya Chandola, Milan Kubicki, Mowpriya Das, Andrea Sette, Domenico Corona, Maurizia Palummo, Letizia Chiodo, René Schöder, Preeti Chahar, Benjamin Fuhrmann, Jonathan Engelhardt, Oskar Düren, Dorothee S. Rosenzweig, Paul Bakos, Kai‐Luis Jakob, Mario Dähne, Conor Hogan, Norbert Esser, Frank Glorius

**Affiliations:** ^1^ Technische Universität Berlin Institut für Physik und Astronomie Hardenbergstraße 36 10623 Berlin Germany; ^2^ Organisch‐Chemisches Institut, Universität Münster Corrensstraße 36 48149 Münster Germany; ^3^ Helmholtz‐Zentrum Berlin für Materialien und Energie GmbH 14109 Berlin Germany; ^4^ Dipartimento di Fisica Università di Roma ‘Tor Vergata' Via della Ricerca Scientifica 1 Rome 00133 Italy; ^5^ CNR Centro S3 CNR‐Istituto Nanoscienze Modena 41125 Italy; ^6^ Department of Engineering Università Campus Bio‐Medico Rome 00128 Italy; ^7^ CNR‐Istituto di Struttura della Materia (CNR‐ISM) Via del Fosso del Cavaliere 100 Rome 00133 Italy

**Keywords:** Monolayers, N‐Heterocyclic carbenes, Organic–inorganic hybrid materials, Semiconductors, Surface functionalization

## Abstract

N‐Heterocyclic carbenes (NHCs) are established ligands for various surfaces, known for their strong binding, ability to form self‐assembled monolayers, and modular structure. However, semiconductor surface modification with NHCs is still in its infancy despite its technological importance. Although previous studies focused on silicon, III‐V compound semiconductors offer direct bandgaps and high electron mobilities, making them ideal for (opto‐)electronics. This study examines the adsorption of different NHCs on GaAs, a prototypical III‐V material, using scanning tunneling microscopy, density functional theory, X‐ray photoelectron spectroscopy, low‐energy electron diffraction, and reflectance anisotropy spectroscopy. Covalent binding to the surface and the formation of well‐ordered NHC monolayers are observed, along with exceptionally large work function reductions. The unique structural features of NHCs enable precise control over film structure, ordering, and electronic properties.

## Graphical abstract

The functionalization of GaAs, a model III‐V semiconductor, with N‐heterocyclic carbenes is demonstrated in a multidisciplinary experimental and theoretical approach. In this way, ordered monolayers consisting of chains of molecules binding covalently to the surface are obtained, which are found to strongly modify the surface properties as evident from large work function reductions.

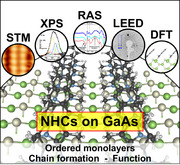

## Introduction

Throughout the past few decades, the demand for functional, high‐performing semiconducting materials has been transformative in the modern world. Gallium arsenide (GaAs) and other III‐V compound semiconductors are among the most important materials in this field with numerous applications, in particular in (opto‐)electronics due to their many advantages, like high electron mobility and direct bandgap.^[^ ^1^, ^2^ ^]^ In this regard, III‐V nanowires have emerged as particularly powerful tools, also allowing a direct integration on low‐cost silicon substrates and a combination of III‐V photonics with silicon electronics.^[^ ^3^, ^4^, ^5^ ^]^

Moreover, hybrid materials comprising both organic and inorganic components offer the potential to add novel or improved functionality to (opto‐)electronic devices.^[^ ^6^ ^]^ For example, hybrid inorganic–organic solar cells have several advantages over traditional inorganic solar cells, such as solution processability and lower production costs.^[^ ^7^ ^]^

An important issue for GaAs‐based devices is surface passivation, as the native oxide layer leads to charge trapping and is unstable under ambient conditions, thus limiting the efficiency of devices.^[^ ^8^, ^9^, ^10^ ^]^ Surface or interface engineering is also important for hybrid III‐V–organic devices, where the presence of defects at the interface often limits the performance.^[^ ^7^ ^]^

Another key point is the energy‐level alignment at the interface, which depends on the interface dipole. The latter can be tuned, e.g., by changing the work function or by inserting a (mono‐)layer of organic donor or acceptor molecules as an interlayer in between the inorganic substrate and further (in‐)organic layers.^[^ ^11^, ^12^ ^]^ At least one material with a low work function is also required for many (opto‐)electronic devices to ensure electron injection or collection at the interface.^[^ ^13^ ^]^

Thus, developing new strategies to tune and modify GaAs surfaces is highly desirable and would be well‐regarded as an important advancement in the field. Surface functionalization with organic molecular modifiers offers a promising strategy.

**Figure 1:**
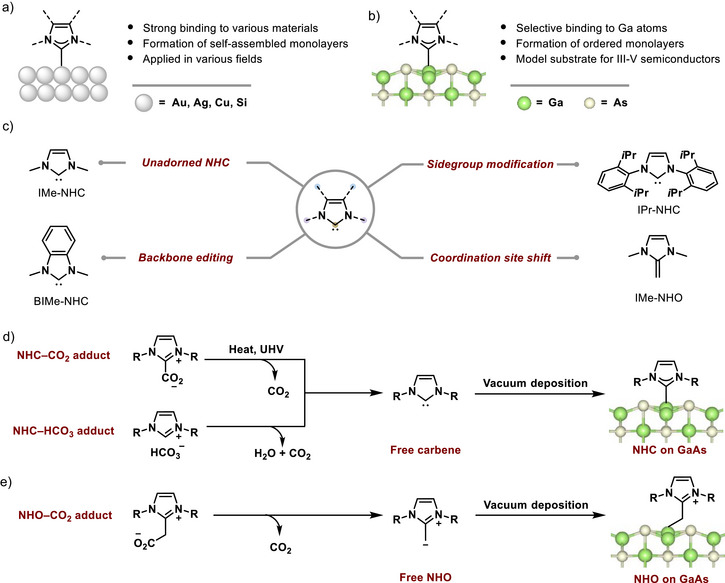
a) NHCs on different elements, a widely investigated and applied system. b) NHCs on GaAs (this work). c) The three NHCs and one NHO applied in this work, as derived from the core‐NHC motif. d,e) Precursors and the UHV evaporation process used here for d) NHC and e) NHO deposition.

N‐Heterocyclic carbenes (NHCs) constitute a well‐established class of organic compounds.^[^ ^14^, ^15^, ^16^ ^]^ The advancements since their discovery have not only propelled the field of organometallic chemistry, but NHCs have also become established modifiers in the field of surface chemistry.^[^ ^17^, ^18^, ^19^, ^20^, ^21^, ^22^, ^23^, ^24^, ^25^, ^26^, ^27^ ^]^ The popularity of NHCs as surface modifiers stems from their many advantageous features, including strong binding to various species, ability to form self‐assembled monolayers, and their modular structure (Figure 1a,c).^[^ ^14^ ^]^ Moreover, NHC layers may also act as anchors for further functionalization or for the growth of further (in‐)organic layers.^[^ ^28^ ^]^ Similarly, related species like N‐heterocyclic olefins (NHOs) and imines can also be used as molecular modifiers for tailoring surface properties.^[^ ^29^, ^30^, ^31^, ^32^, ^33^, ^34^, ^35^ ^]^ NHCs are ubiquitous surface modifiers for noble metals, but recently they have been adopted for other materials such as metal oxides and semiconductors.^[^ ^36^, ^37^, ^38^, ^39^, ^40^, ^41^, ^42^, ^43^, ^44^, ^45^, ^46^, ^47^, ^48^, ^49^, ^50^, ^51^ ^]^ Beyond material versatility, NHC‐modified surfaces are finding applications across diverse fields, e.g., in catalysis and biosensing.^[^ ^52^, ^53^, ^54^, ^55^, ^56^, ^57^, ^58^, ^59^, ^60^, ^61^, ^62^, ^63^, ^64^ ^]^ Furthermore, we recently demonstrated the modification of silicon surfaces with NHCs^[^ ^65^, ^66^ ^]^ and NHOs,^[^ ^34^ ^]^ with both molecule classes covalently binding to Si atoms, resulting in a stable monolayer formation with a significant reduction in the work function. To the best of our knowledge, there have been no reports on a modification of GaAs with NHCs or NHOs.

Here, we present a comprehensive study of the binding and growth of structurally different NHC and NHO molecules on GaAs(110) via scanning tunneling microscopy (STM), density functional theory (DFT), X‐ray photoemission spectroscopy (XPS), low‐energy electron diffraction (LEED), and reflectance anisotropy spectroscopy (RAS) (Figure 1b). The (110) surface is also the preferred orientation of the sidewall facets of III‐V nanowires,^[^ ^4^ ^]^ underpinning the importance of its functionalization or passivation for device applications. Furthermore, GaAs(110) can be seen as a model surface for other III‐V and II‐VI semiconductors forming in the zincblende crystal structure.

Different molecular modifiers were meticulously selected to investigate the effects of structural modulation of the core NHC motif on the adsorption geometry, surface‐assembly behavior, and electronic properties of the substrate (Figure 1c). Herein, two types of stable precursors were used for deposition of the respective NHC and NHO molecules, ${\mathrm{CO}}_{2}$ adducts and bicarbonate (${\mathrm{HCO}}_{3}$) adducts. They both result in a clean deposition of the respective molecule via controlled heating in ultra‐high vacuum (UHV) conditions (Figure 1d,e), which is proven by the XPS overview scans presented in Section S13.

## Results and Discussion

**Figure 2:**
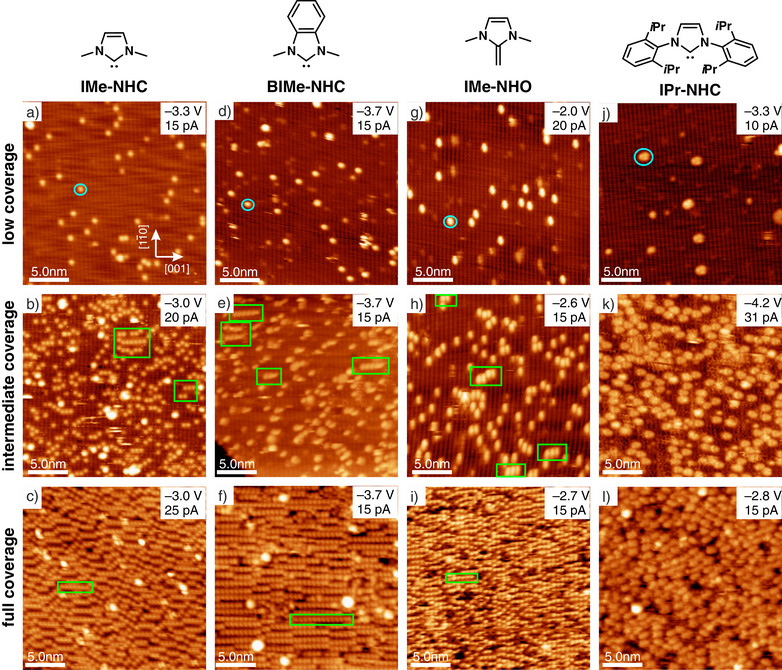
Overview STM images of low, intermediate, and full monolayer coverages of all investigated molecules on GaAs(110). The STM images within each row show representative examples for the respective coverage range, while the exact coverage varies. Isolated molecules and chains are indicated by blue circles and green boxes, respectively. All tunneling conditions (sample voltage $V_{T}$ and tunneling current $I_{T}$) are given in the images.

As mentioned above, the four different molecules shown in Figure 1c were deposited on GaAs to investigate the influence of 1) different sidegroups, 2) backbone modification, and 3) terminal carbon coordination. Figure 2 shows overview STM images for low, intermediate, and full monolayer coverages of all molecules. This, along with the XPS results for the C 1s and N 1s core levels presented in Section S14, confirms the successful modification of the GaAs surface.

At low molecular coverages (top row in Figure 2), isolated molecules appear as bright spots randomly dispersed across the surface with no evident clustering (examples of the respective molecules are indicated by blue circles). In between, the vertically running Ga–As atomic rows of the substrate surface are still visible (a detailed description of the GaAs(110) surface is given in Section S5). As expected, IPr‐NHC with its bulky sidegroups appears considerably larger as compared to the other smaller molecules with methyl sidegroups.

At intermediate coverages (central row of Figure 2), many isolated molecules are still observed. However, for the molecules with methyl sidegroups, short chains are observed (examples are indicated in green). These molecule chains are aligned along the [001] direction, i.e., perpendicular to the orientation of the Ga–As rows of the substrate, with one molecule adsorbing on every Ga–As row. For IPr‐NHC, in contrast, such a chain formation is not observed.

In the STM images at full coverage (bottom row in Figure 2), again a similar behavior is observed for IMe‐NHC, BIMe‐NHC, and IMe‐NHO in the form of relatively well‐ordered monolayers, with the highest grade of ordering found for BIMe‐NHC. They are characterized by chains of NHC molecules exhibiting the same orientation and intermolecular distances as those found for intermediate coverages. Thus, the latter are precursors of the monolayers, and their formation continues until the ordered monolayers are obtained. In stark contrast, a disordered monolayer is observed for IPr‐NHC without any indication of a chain formation.

This behavior is astounding, as IMe‐NHC, BIMe‐NHC, and IMe‐NHO strongly differ from each other, e.g., in terms of their structural and electronic properties. Their common feature is the presence of methyl sidegroups, while IPr exhibits large diisopropylphenyl sidegroups. It is thus evident that the sidegroup and backbone modification play an important role for the growth and assembly behavior.

### Adsorption Geometry and Chain Formation

In order to investigate the driving force behind the formation of these chains in the case of the molecules containing smaller side groups, we performed DFT calculations for pairs of NHC or NHO molecules in a large simulation cell for different geometries and separations.

**Figure 3:**
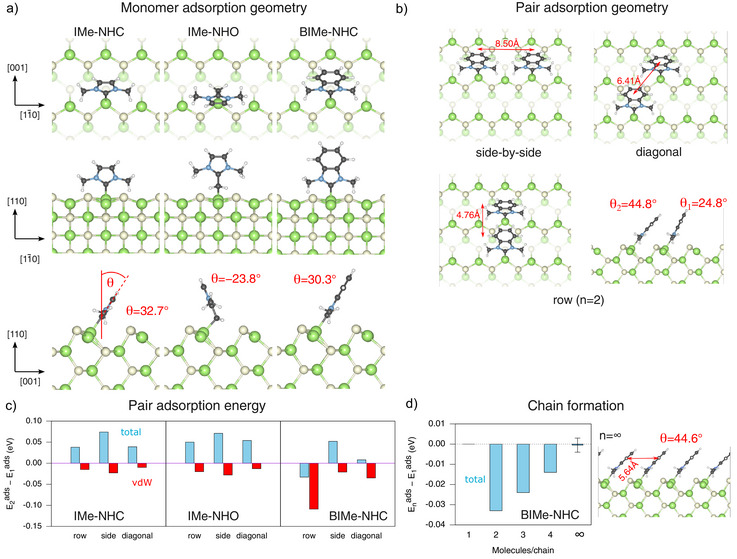
a) Adsorption geometries of single molecules. Planar tilt angles with respect to the surface normal are indicated. b) Pair adsorption geometries for BIMe‐NHC. The π–π distances are indicated. c) Pair adsorption energies $E_{2}^{\mathrm{ads}}$ (per molecule), relative to those of isolated monomers $E_{1}^{\mathrm{ads}}$. Blue: total $E^{\mathrm{ads}}$. Red: vdW contribution $E_{\mathrm{vdW}}^{\mathrm{ads}}$. d) Adsorption energy (per molecule) for chain formation, relative to the monomer adsorption energy. The ideal n=∞ geometry is indicated on the right.

As a reference, the monomer adsorption geometries for ‘isolated’ molecules are first considered, as shown in Figure 3a for the three molecules with methyl sidegroups. They all bind to the Ga surface atoms by forming a covalent bond, resulting in an upright, but tilted geometry (see also Section S6). Binding to As surface atoms is not favored. The orientation of the molecules is such that the central heterocyclic ring aligns along the [1$\bar{1}$0] direction, i.e., the direction of the Ga–As rows. The Ga atoms binding to the molecules are slightly displaced outward, thus locally lifting the relaxation of the GaAs(110) surface and adopting an $sp^{3}$‐like configuration. This also determines the tilt angles for isolated molecules at around 30 with respect to the surface normal. The Ga–C bond lengths are 2.09 Å (IMe‐NHC) and 2.10 Å (BIMe‐NHC). IMe‐NHO is a peculiarity because of its additional alkylidene unit: it adopts a more vertical adsorption geometry with a slight tilt in the opposite direction in order to allow its terminating carbon atom to bind to the Ga atom in an $sp^{3}$‐like geometry. Nonetheless, the Ga–C bond length is consistent, at 2.10 Å. The calculated adsorption energies are listed in Table S1 and lie between −1.91  and −2.07 eV. These energies include a subtle balance between strain, van der Waals (vdW) coupling, and covalent bond formation. For instance, the tilted geometry of IMe‐NHC (and BIMe‐NHC) allows the molecule to maximize their vdW coupling with the chelating surface As atoms. Moreover, the more ionic nature of the Ga–C bond likely lessens the distinction between the electronic properties (carbon hybridization) of the NHCs and the NHO with regard to the adsorption energy.

To investigate further the Ga–NHC and Ga–NHO binding modes, we performed voronoi deformation density (VDD)^[^ ^67^ ^]^ and charge density difference (CDD) analyses. The results are reported in Figures S11 and S12. The VDD analysis indicates a very similar electron transfer to the substrate for IMe‐NHC (0.64e) and IMe‐NHO (0.63e), despite the very different electronic properties of the binding C atom. The donated charge is almost completely localized in the topmost Ga–As layer but is distributed among several Ga and As atoms near the bonding site. A significant charge redistribution (polarization) is also observed within the molecules themselves (see Figure S12). Both of these contribute to the formation of distinct surface dipoles.

**Figure 4:**
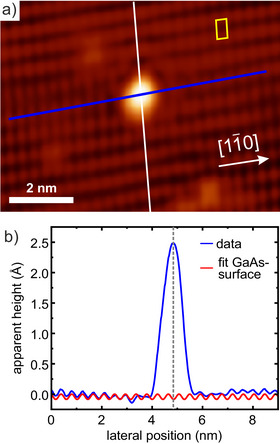
a) Atomically resolved filled states STM image ($V_{T}=-3.4$ V; $I_{T}=10$ pA) of an isolated BIMe‐NHC molecule on GaAs(110) and b) corresponding height profile (blue) along the line indicated in (a). The white line indicates the positions of Ga atoms, the red fit curve those of the As atoms. A unit cell of the GaAs(110) surface is shown in yellow.

Experimentally, the adsorption site can be best determined from STM images at low molecular coverages, as they offer atomic resolution on both the substrate and the isolated molecules. Such images are shown in Figure 4 for BIMe‐NHC and in Section S8 for the other molecules. Indeed, the images perfectly support the theoretical prediction of the adsorption sites: in filled state images, as shown in Figure 4a, the bright rows in the background correspond to As atoms. As the molecules appear centered in between two neighboring As atoms of a Ga–As atomic row, the STM results confirm that the adsorption occurs at the Ga atoms.

Now we turn our attention to pairwise adsorption in the nearest possible “diagonal,” “side‐by‐side,” and “row” geometries, as illustrated in Figure 3b and Figure S8 for BIMe‐NHC (corresponding geometries for IMe‐NHC and IMe‐NHO are reported in Figures S6 and S7, respectively). The total adsorption energy per molecule and vdW component for IMe‐NHC, IMe‐NHO, and BIMe‐NHC pair adsorption, relative to that of the isolated monomer, are plotted in Figure 3c. This figure demonstrates that, despite an attractive vdW component for all three geometries, the effect of strain and steric repulsion renders the total energies positive for all IMe‐NHC and IMe‐NHO configurations (i.e., at low coverages the molecules prefer to remain isolated). Instead, BIMe‐NHC shows a net negative energy for a row alignment, while the diagonal geometry is only slightly disfavored. This difference can be attributed to a very large vdW contribution to the total energy, which is maximized via the asymmetric tilted geometry evident in Figure 3b for a short BIMe‐NHC row. In fact, no noticeable deviation from the isolated geometry is observed for IMe‐NHC or IMe‐NHO, as shown in Figure S5. Note that the large π–π distances (> 4.7 Å; see Figure 3) tend to rule out an attractive π–π coupling.

At intermediate coverages, some short chains are also observed for IMe‐NHC and IMe‐NHO, in apparent contradiction with the positive (relative) pair adsorption energies in Figure 3c. However, also the local coverage is important: the impinging molecules have to adsorb somewhere, and the lateral pressure and diffusion barriers play increasingly relevant roles. What is notable for these molecules is the similar energies of row and diagonal geometries, in contrast to the side geometry. Thus, short chains with kinks are expected to form when the local coverage precludes the favored isolated geometry, consistent with the observations in Figure 2.

To cast light on this, we performed a simple DFT study of short (n=1…4) and infinite (n=∞) BIMe‐NHC chains and calculated the relative adsorption energy per molecule $E_{n}^{\mathrm{ads}}-E_{1}^{\mathrm{ads}}$ for increasing chain length. As shown in Figure 3d and Figure S9, we find that the relative energy tends to decrease with increasing n, implying that longer chains will eventually fall in competition with the small (albeit positive) energy for the diagonal geometry (Figure 3c). Although the analysis is limited (our treatment of the vdW interaction is semi‐empirical), it predicts that chains of ∼5–10 molecules could form before kinks appear, in general agreement with the experimental observations. We note that the formation of isolated linear chains at low coverage is anyway inhibited by the lack of barrier‐free diffusion on the surface.

The absence of a chain formation for IPr‐NHC can be explained by a different rotated adsorption geometry. Our DFT calculations for isolated IPr‐NHC molecules reveal large differences when compared to the other molecules with methyl sidegroups (see Section S6 and Figure S10). Two different geometries of similar energy are found: in the more stable geometry 1, a Ga–C bond is clearly formed ($d_{\mathrm{Ga}}-C=2.3$ Å), while the other adsorption geometry 2 appears more physisorbed in nature ($d_{\mathrm{Ga}}-C=3.9$ Å). This is also confirmed by STM results (see Section S8 and Figure S15). For both geometries the central heterocyclic ring is not aligned along the [1$\bar{1}$0] direction. This orientation together with the large sidegroups sterically hinders a strong intermolecular vdW and/or π‐stacking interaction, so that a chain formation is unfavored in that case.

### Monolayer Structure

**Figure 5:**
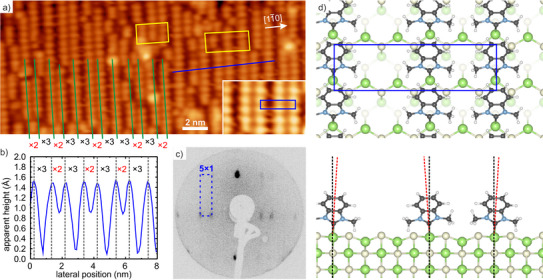
a) High resolution STM image ($V_{T}=-3.6$ V; $I_{T}=15$ pA) of the BIMe‐NHC monolayer. Some BIMe‐NHC chains with alternating distances of two and three times the lattice constant are highlighted by green lines, the yellow boxes indicate regions where other arrangements of the chains are found. The inset shows an enlarged view with a (5×1) unit cell indicated. b) Height profile along the blue line in (a); distances between neighboring chains are indicated. c) LEED diffraction pattern for a kinetic energy *E* 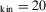 eV showing the (5×1) periodicity of the BIMe‐NHC monolayer. d) DFT geometry of the of (5×1) BIMe‐NHC monolayer in top and side view. An outward bending of the molecules is visible as indicated by the black and red dashed lines.

Now, the detailed structure of the monolayers is discussed. Figure 5 shows high‐resolution STM data as well as LEED and DFT results for the BIMe‐NHC monolayer.

As mentioned above, the molecular chains run along the [001] direction, i.e., perpendicular to the Ga–As rows of the substrate. Here, a periodicity of 0.57 nm is found corresponding to the lattice constant $a_{[}001]$ in this direction, demonstrating that the molecules adsorb on every Ga–As row. In the perpendicular $\left[1\bar{1}0\right]$ direction, in contrast, the distances between neighboring molecular chains are larger and furthermore often vary alternately between a shorter and a larger distance, as indicated in Figure 5a by the green lines and clearly visible in the height profile presented in Figure 5b. With some exceptions, an arrangement with two molecular chains with closer distance that are separated from the following pair of molecular chains by a larger distance is found. The closer distance thereby corresponds to about twice the lattice constant $a_{[}1\bar{1}0]$ in that direction, while the larger separation amounts to about 3$a_{[}1\bar{1}0]$ (indicated by ×2 and ×3, respectively). The overall periodicity of such an arrangement can thus be described as (5×1), a corresponding unit cell is indicated in the inset in Figure 5a. Further evidence for the high order of the BIMe‐NHC monolayer comes from LEED measurements, as shown in Figure 5c, where the diffraction pattern directly shows the (5×1) periodicity. Despite the high grade of ordering, deviations from the perfect ordering are also observed. For example, displacements along the molecular rows occur, and occasionally also three neighboring rows with ×2 or ×3 distance are observed (see, e.g., yellow boxes in Figure 5a).

The reason for these alternating ×2 and ×3 separations can be understood by carefully inspecting the calculated geometric structure of the (5×1) monolayer, as shown in Figure 5d. Adjacent molecules clearly exhibit an outward bending as indicated by the red dashed lines in the side view. This allows the strain to be lowered at the close ×2 distance and renders alternating ×2 and ×3 separations a good compromise between coverage maximization and energy minimization. The outward bending of the BIMe‐NHC molecules can even be observed in our STM data: As discussed in Section S9, the large separations are always found to be slightly smaller as compared to the nominal value of 3$a_{[}1\bar{1}0]$, while the smaller separations are always slightly larger as compared to 2$a_{[}1\bar{1}0]$.

Although the grade of ordering and the chain length is highest for BIMe‐NHC, a similar arrangement into molecular chains with ×2 and ×3 separations between neighboring chains is also observed for the other molecules with methyl sidegroups (see Figure 2c,i). This leads also to very similar molecular coverages for these molecules around 0.35±0.03 molecules per GaAs(110) unit cell, as determined from STM and XPS data (see Section S10). This value is slightly smaller as compared to the theoretical coverage of 0.40 molecules per GaAs(110) unit cell for a perfect (5×1) monolayer. For IPr‐NHC, a much lower molecular coverage of 0.14±0.03 molecules per GaAs(110) unit cell is obtained related to its larger size and the formation of the disordered monolayer.

### Optical Characterization of NHC Monolayers

**Figure 6:**
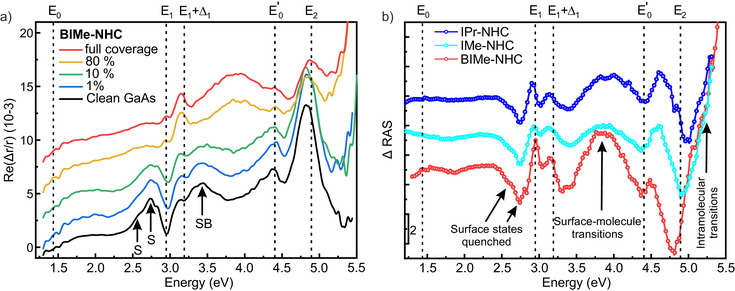
a) RAS measurements for a series of BIMe‐NHC coverages (given in percentage of the full monolayer coverage). Surface state transitions are denoted by S and transitions between surface and surface‐perturbed bulk states by SB. b) Difference between clean and molecule‐saturated surface Δ RAS for BIMe‐NHC, IMe‐NHC, and IPr‐NHC monolayers.

In Figure 6a, we present reflectance anisotropy spectroscopy (RAS) measurements for the GaAs(110) surface before and after adsorption of BIMe‐NHC. RAS is a non‐destructive optical probe that is particularly sensitive to local features in the surface geometry and electronic structure.^[^ ^68^ ^]^ For organic adlayers, it can provide information about the molecular orientation and the modification or quenching of surface states.^[^ ^69^, ^70^, ^71^ ^]^ To our knowledge, it has not been applied to the characterization of NHCs or NHOs on surfaces.

RAS of clean GaAs(110) has been widely studied and features peaks from surface state transitions around 2.6 eV (S), a broad structure at 3.4 eV related to transitions between surface and surface‐perturbed bulk states (SB), and derivative lineshapes (*E* 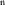) associated with critical points in the bulk GaAs bandstructure.^[^ ^72^, ^73^, ^74^ ^]^ The S features are known to arise from electronic transitions between the As filled states (lone pairs) and the Ga empty states (dangling bonds) of the relaxed (110) surface.^[^ ^72^, ^73^ ^]^

Upon BIMe‐NHC adsorption, the *S* and SB signals are clearly quenched, providing further evidence for chemical bonding to the Ga atoms as proposed from the STM, XPS, and DFT analysis.

In addition, a strong broad signal is identified at 3.8 eV that increases with deposition time. The positive sign of the signal indicates optical transitions along the [$1\bar{1}0$] direction, i.e., along the Ga–As chains. Moreover, a strong increase of the optical anisotropy is observed above 5 eV, close to the upper limit of the RAS setup (5.4 eV). We enhance the contribution by plotting the relative change in the RAS with respect to that of the clean surface, the so‐called Δ RAS, as shown in Figure 6b. The Δ RAS data show that the molecule‐induced signals are also present for IMe‐NHC and IPr‐NHC, but to a lesser extent.

Although it is natural to interpret these positive Δ RAS signals as deriving from optical transitions localized within the molecules, our beyond‐DFT (GW‐BSE approach) calculations on gas‐phase molecules (Section S11) indicate that the optical gap is not lower than 4.8 eV, and thus intramolecular excitations may only explain the high‐energy signal above 5 eV.

**Figure 7:**
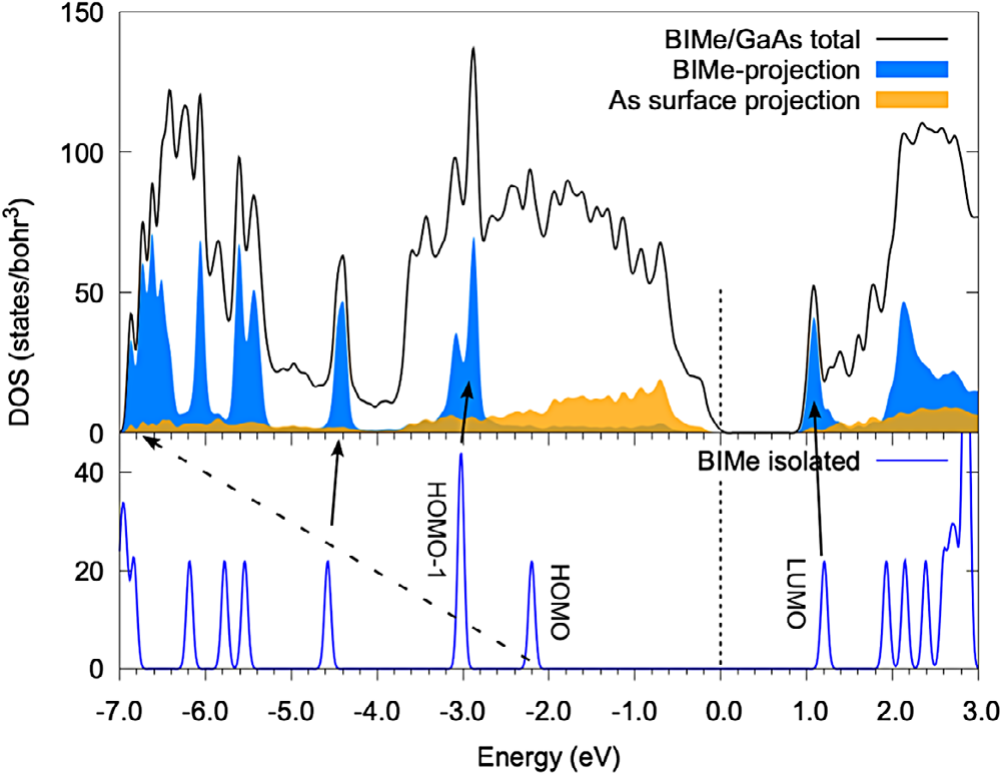
Density of states (DOS) of the BIMe‐NHC/GaAs(110) slab and projected components. The DOS of the gas‐phase BIMe‐NHC molecule is plotted underneath.

To understand the signal at 3.8 eV, we carried out DFT calculations of the (5×1)‐BIMe‐NHC monolayer and analyzed the electronic properties. Due to the large system size, the DFT‐PBE functional is used and thus an underestimation of energy levels and gaps is expected. The top panel of Figure 7 shows the total DOS as well as the projected DOS on the BIMe‐NHC molecule and surface As atoms, respectively. The bottom panel shows the DOS of the gas‐phase BIMe‐NHC molecule. By performing a molecular‐projected DOS analysis,^[^ ^75^ ^]^ specific peaks in the BIMe‐NHC projected DOS can be related back to specific energy levels in the gas‐phase molecule. Hence, the HOMO, which is associated with the lone pair of the carbene C atom, hybridizes with the Ga surface state upon formation of the C–Ga bond. The resulting bonding and antibonding orbitals are shifted far from the gap (the final position of the former is indicated by the dashed arrow in Figure 7). Instead, the LUMO and HOMO‐1 levels (and others) remain relatively unperturbed. From this analysis, we identify the sharp peak in the GaAs/BIMe‐NHC DOS at 1.1 eV as coming from the LUMO of the molecule.

The presence of molecular levels close to the GaAs conduction band minimum makes it likely that surface‐molecule optical transitions are responsible for the observed broad peak at 3.8 eV. For instance, a possible candidate (allowing for energy gap corrections) involves transitions between filled As surface dangling bonds that are not quenched by molecular adsorption and the molecular LUMO or LUMO+1 states, which are spatially close enough to the surface to overlap with the As dangling bonds. The relatively larger strength of the BIMe‐NHC signal, with respect to IMe‐NHC or IPr‐NHC, is most likely due to the improved ordering.

### Work Function Reduction

In order to demonstrate that the NHC and NHO monolayers are able to directly modify the properties of the GaAs surface, the change in work function ΔW occurring upon formation of the monolayers is determined by measuring the secondary electron (SE) onsets for both the clean GaAs surface and the formed NHC and NHO monolayers using XPS. The experimental data are presented in Section S12, the results are summarized in Table 1.

**Table 1:** Experimentally determined work function changes ΔW for BIMe‐NHC, IMe‐NHC, IMe‐NHO, and IPr‐NHC monolayers on GaAs.

Molecule	Work function change ΔW (eV)
BIMe‐NHC	−2.00±0.10
IMe‐NHC	−2.06±0.10
IMe‐NHO	−2.31±0.10
IPr‐NHC	−1.91±0.10

For all molecules, extraordinarily large work function reductions of about 1.9–2.3 eV are found. The observed changes are thereby even larger than those observed for NHC and NHO molecules on other surfaces such as gold or silicon.^[^ ^34^, ^65^, ^76^ ^]^ They originate first from a charge transfer from the molecules to the surface, resulting in an interface dipole, and second from a dipole formation within the molecules, as discussed, e.g., in Das et al.^[^ ^34^ ^]^

The largest reduction of −2.31 eV is found for IMe‐NHO. Partly, this is related to a larger initial work function of the clean GaAs surface found in this case (see Section S12 for more details). However, this can only explain part of the large work function reduction, as also the final value for the SE onset is lowest for IMe‐NHO. As the IMe‐NHO adsorption geometry is closest to vertical for the molecules with methyl sidegroups, it may be expected that also the vertical component of the internal molecular dipole moment is largest for this molecule, leading to the observed extremely low final work function.

Due to their larger tilt angles and the resulting smaller vertical components of their internal molecular dipole moments, slightly smaller work function reductions are found for IMe‐NHC and BIMe‐NHC.

On the other hand, the lowest value in ΔW of −1.91 eV observed for IPr is the result of the lower surface coverage due to its large size. In this case, the lower surface coverage (by a factor of about three; see above) is compensated by more options for charge redistribution within the larger molecule, so that still a remarkable reduction of the work function is obtained.

## Conclusion

In a comprehensive STM, DFT, XPS, RAS, and LEED study, NHC and NHO adsorption on GaAs was investigated. Four different molecules were used to systematically investigate the influence of varying side groups, backbone modifications, and the comparison between NHC and NHO molecular groups. For the molecules with methyl sidegroups, we demonstrate the growth of well‐ordered monolayers consisting of chains of NHC or NHO molecules. Strain between neighboring chains finally determines the arrangement in the monolayer. Moreover, we demonstrate that extraordinarily large work function reductions occur upon monolayer formation for all investigated molecules. This fascinating property may allow a controlled band‐offset engineering to further inorganic or organic overlayers. The study is completed by an optical characterization using RAS for the first time to investigate NHCs.

The NHC and NHO monolayers on GaAs developed here and the obtained detailed understanding of the adsorption and growth open the door towards a tailor‐made modification or functionalization of III‐V semiconductor surfaces.

## Supporting Information

For synthesis, experimental and computational details, supplementary figures and tables, see Supporting Information. Supporting Information is available from the Wiley Online Library or from the authors.

## Conflict of Interests

The authors declare no conflict of interest.

## Supporting information

Supporting Information

## Data Availability

The data that support the findings of this study are available from the corresponding authors upon reasonable request.
