# Supplementary material for: N‐Heterocyclic Carbenes on a III‐V Semiconductor: From Chain Formation to Ordered Monolayers
Source: Angew Chem Int Ed Engl. 2025 Sep 22;64(46):e202511094. doi: 10.1002/anie.202511094 (PMC12603996; doi:10.1002/anie.202511094)
Supplement: Supplementary file 1 — Supporting Information [file ANIE-64-e202511094-s001.pdf]

# Supporting Information
# N-Heterocyclic Carbenes on a III-V Semiconductor: from Chain Formation to Ordered Monolayers

Martin Franz, Ankita Das, Sandhya Chandola, Milan Kubicki, Mowpriya Das, Andrea Sette, Domenico Corona, Maurizia Palummo, Letizia Chiodo, René Schöder, Preeti Chahar, Benjamin Fuhrmann, Jonathan Engelhardt, Oskar Düren, Dorothee S. Rosenzweig, Paul Bakos, Kai-Luis Jakob, Mario Dähne, Conor Hogan,* Norbert Esser,* Frank Glorius*

# Contents

1. Experimental Methods 3
1.1. Scanning Tunneling Microscopy . . . 3
1.2. Low-Energy Electron Diffraction . . . 3
1.3. Reflectance Anisotropy Spectroscopy . . . 3
1.4. X-Ray Photoelectron Spectroscopy . . . 3

2. Sample Preparation 3

3. Computational Details 4

4. Synthesis 4
4.1. General Information . . . 4
4.2. Synthesis of NHC · $CO_2$ Adducts . . . 4
4.3. Synthesis of NHO · $CO_2$ Adduct . . . 5
4.4. Synthesis of NHC · $HCO_3$ Adduct . . . 6

5. The GaAs(110) Surface 13

6. Adsorption Geometries from DFT 15
6.1. BIMe-NHC, IMe-NHC, and IMe-NHO . . . 15
6.2. IPr-NHC . . . 19

7. Charge Density Analysis from DFT 20

8. Determination of the Adsorption Site from STM 22
8.1. BIMe-NHC, IMe-NHC, and IMe-NHO . . . 22
8.2. IPr-NHC . . . 22

9. Detailed Monolayer Structure 24

10. Coverage Determination 24

11. Optical Properties Calculations 25

12. Determination of the Work Function Change 26

13. Overview XPS Spectra 27

14. C 1*s* and N 1*s* Core Levels 27

15. Ga 3*d* and As 3*d* Core Levels 27

References 31

# 1. Experimental Methods

For the experiments, two separate ultra-high vacuum (UHV) chamber systems hosting comparable sample preparation facilities were employed. One chamber system was used for scanning tunneling microscopy (STM), low-energy electron diffraction (LEED), and reflectance anisotropy spectroscopy (RAS) measurements. Here, the base pressure was in the $10^{-9}$ to $10^{-8}$ Pa range and did not exceed $3{\times}10^{-7}$ Pa during the whole preparation process. A second UHV chamber system (base pressure $10^{-8}$ Pa) was used for X-ray photoelectron spectroscopy (XPS). All experiments in this work were performed at room temperature.

## 1.1. Scanning Tunneling Microscopy

For the STM experiments, a home-built STM setup with a commercial control electronics (Nanonis) operating in constant-current mode was used. The tips were electrochemically etched from W wires in our lab and cleaned *in-situ* by electron bombardment prior to usage.

## 1.2. Low-Energy Electron Diffraction

LEED measurements were performed *in-situ* to check the quality and to determine the surface periodicity of both clean and fully covered GaAs(110) samples. Therefore, a Vacuum Science Instruments ErLeed 150 optics and a SPECS 3000D control electronics were used.

## 1.3. Reflectance Anisotropy Spectroscopy

The RAS setup is capable of measuring in the spectral range from 1.2 to 5.5 eV. The setup comprises a laser driven light source, a $CaF_2$ photoelastic modulator, $MgF_2$ Rochon polarizers, and a Si photodiode. RAS probes the difference in the reflectance $R$ at near normal incidence of light linearly polarized in two orthogonal crystal axes $x$ and $y$.[1] The RAS signal is defined by

$$\frac{\Delta R}{R} = \frac{R_{\mathrm{x}} - R_{\mathrm{y}}}{R}. \tag{1}$$

All RAS spectra in this work are expressed in terms of the real part of the anisotropy in the reflection coefficients $\Delta r/r$, which is related to the signal by $\Delta R/R = 2\mathrm{Re}(\Delta r/r)$.

## 1.4. X-Ray Photoelectron Spectroscopy

XPS was measured using a hemispherical energy analyzer (SPECS Phoibos 100) and monochromatized Al-$K_\alpha$ X-rays with $h\nu = 1486.7$ eV (SPECS FOCUS 500 Ellipsoidal Crystal Monochromator and X-Ray source SPECS XR50 M) yielding an instrumental resolution of $\sim$0.4 eV. The binding energy scale was calibrated with the Au $4f$ line at 84.0 eV using a Au foil in direct contact with the sample holder as well as the Fermi edge measured on a Mo sample holder.

To determine the change in work function, the secondary electron (SE) for the clean GaAs(110) surfaces as well as for the fully covered samples were measured. To ensure that all SE reach the analyzer, a bias of $-9$ V was applied to the sample. This represents a well-established method[2,3] as has been demonstrated in a variety of studies, e.g. for alkali metal[4–6] or NHC and NHO molecule[7–10] deposition on different substrates.

The core-level spectra were fitted using Voigt line profiles for considering both lifetime and instrumental broadening. Both a constant and a Shirley-type background were used.

Unless otherwise noted, the core-level spectra were measured under an angle of 70° with respect to the surface normal to enhance the surface sensitivity. All work function measurements were performed in normal emission.

# 2. Sample Preparation

The samples were cut from $n$-type GaAs crystals and provided with notches along the intended (110) cleavage plane. Then they were mounted on the sample holders using an electrically conductive silver epoxy (Epoxy technologies epo-tek® H20E). After transferring into the UHV chamber systems, the samples were cleaved using similar cleavage tools in both systems.

The NHC and NHO molecules were deposited using an analogous method as introduced for NHCs in previous studies[7,9,11]. As precursors, $CO_2$ adducts were used for IMe-NHC, IPr-NHC, and IMe-NHO, while $HCO_3$ adducts were used for BIMe-NHC. Their synthesis is described in detail below in Sec. 4. Prior to usage, the adducts were stored under Ar atmosphere in a freezer at –18 °C.

Home-built evaporators with pyrolytic boron nitride crucibles and crucible temperatures in the range from room temperature to 40 °C were used for evaporation. This results in deposition times between a few seconds and several minutes for the investigated low and high coverage samples, respectively. During deposition, the sample was held at room temperature and during the whole sample preparation, the pressure did not exceed $2 \times 10^{-7}$ Pa.

When heated under UHV conditions, the precursors decompose by detaching either $CO_2$ ($CO_2$ adducts) or $CO_2$ and $H_2O$ ($HCO_3$ adducts), so that a molecular beam of free NHC or NHO molecules is obtained. During deposition, we monitor the

partial pressures of all relevant masses using a quadrupole mass spectrometer (QMS) installed in the deposition chamber.[9] Our QMS data show a successful decomposition with solely an increase in the $CO_2$ signal for the $CO_2$ adducts. For the $HCO_3$ adducts, an increase in the $H_2O$ signal is observed additionally, as expected. Thus, we conclude that the adducts remained stable during storage. The deposition of clean NHC and NHO molecules without any residuals from the adducts is additionally proven by the absence of an O 1$s$ signal in XPS overview spectra (see Figure S19).

# 3. Computational Details

Density functional theory (DFT) calculations were performed using the quantum-ESPRESSO code[12] within a planewave (cutoff 50 Ry) and norm-conserving pseudopotential framework. The Perdew-Burke-Ernzerhof (PBE) exchange-correlation functional was used along with the semi-empirical Grimme-D3 van der Waals (vdW) correction including Becke-Johnson damping[13]. The GaAs(110) surface was modelled using periodic supercells containing symmetric slabs with seven atomic layers, separated by 20 Å of vacuum. Molecules were adsorbed on one side of the slab. For DOS calculations, thicker 11-layer slabs were used, with molecules on both sides. Monolayers were modelled using surface unit cells of size $2 \times 1$, $3 \times 1$ and $5 \times 1$. Isolated molecule adsorption was simulated with a larger $5 \times 4$ cell, except for IPr which used a $7 \times 5$ cell. Pairwise adsorption and chain formation were simulated with $5 \times 4$ and $7 \times 5$ cells. The surface Brillouin zone was sampled using the $\Gamma$ point only for $5 \times 4$ and $7 \times 5$ cells, and denser $4 \times 16 \times 1$ meshes for $5 \times 1$ cells. Geometry optimizations were carried out using very tight thresholds of 2.6 meV/Å on the atomic forces. DOS and molecular projected DOS[14] was computed with a dense $8 \times 32 \times 1$ k-point grid. Adsorption energy for $n$ molecules for a fixed surface area is defined as

$$E_{\mathrm{n}}^{\mathrm{ads}} = (E_{\mathrm{n}}^{\mathrm{GaAs+mol}} - E^{\mathrm{GaAs}} - nE^{\mathrm{mol}})/n \qquad (2)$$

where the terms on the right hand side are the total energies of slab + $n$ molecules, clean slab, and gas phase molecule, respectively. Images of geometries were prepared with VESTA.[15] Voronoi deformation density (VDD) charges[16] were computed using the critic2 code.[17]

Excited state electronic levels and optical spectra of the isolated BIMe-NHC molecule were computed with the MOLGW code.[18] Single quasiparticle levels were computed using the GW approximation for the self energy. In particular we used a partially self-consistent G$n$W$n$ method starting from B3LYP eigenvalues and eigenstates, which eliminates quite well the starting-point dependence. The optical spectra including local field and excitonic effects were computed by solving the Bethe-Salpeter equation starting from the quasiparticle bandstructure. The aug-cc-pVTZ basis set was used.[19] We estimate this approach yields an error of up to 0.5 eV in the GW gap and up to 0.3 eV in the position of the optical gap.

# 4. Synthesis

## 4.1. General Information

All reactions were carried out in oven-dried glassware with oven-dried Teflon-coated magnetic stir bars. Dry solvents were either taken from a solvent purification system (HPLC grade, dried over activated alumina columns) or purchased from Acros Organics, Sigma-Aldrich or Carl Roth (stored over activated molecular sieves).

**$^1$H- and $^{13}$C-NMR spectra** were recorded on a Bruker AV 400 at room temperature. Chemical shifts ($\delta$) are given in ppm. The residual solvent signals were used as references and the chemical shifts converted to the TMS scale (MeOD: $\delta$H = 4.87 ppm, $\delta$C = 49.00 ppm; $CD_2Cl_2$: $\delta$H = 5.32 ppm, $\delta$C = 54.00 ppm; $D_2O$: $\delta$H = 4.79 ppm,). All the NMRs were processed using Mestrenova 14 applying standard phase and baseline corrections. Coupling constants (J) are quoted in Hz.

**High resolution mass spectra (HRMS)** were recorded on a Thermo Scientific Exploris 120 Electrospray Orbitrap in electrospray ionizsation mode (ESI). ESI spectra show relative abundance after normalization against maximum signal intensity level (NL) in dependence of $m/z$.

**Direct inlet electron impact mass spectra with temperature profile (Direct inlet EIMS)** for extimating the evaporation temperature of the carbene precursor were recorded on a Thermo Scientific TSQ 7000: The solid material was placed in a clean crucible, transferred into the high vacuum of the spectrometer and heated from room temperature to 400 °C to follow the evaporation of the "free" NHC.

## 4.2. Synthesis of NHC · $CO_2$ Adducts

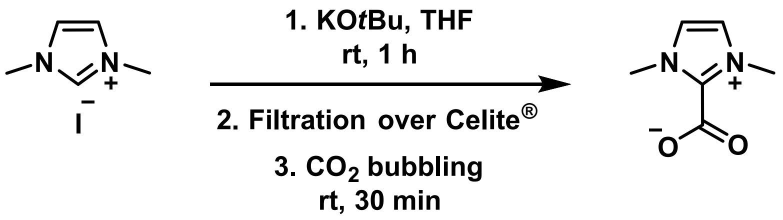


**1,3-dimethyl-1H-imidazol-3-ium-2-carboxylate (IMe-NHC · $CO_2$):** The compound was synthesized following a modified literature procedure.[20] To a flame dried Schlenk flask under argon was added 1,3-dimethyl-1H-imidazol-3-ium

iodide (1.0 equiv., 1 mmol) and KO*t*Bu (1.5 equiv., 1.5 mmol ). Dry THF (10 mL) was then added to the mixture and the suspension was stirred for 1 h at room temperature. The suspension was subsequently filtered under argon through a Celite® pad and carbon dioxide dried via conc. sulphuric acid was bubbled through the solution for 30 min. During this time, a white solid precipitate was observed which was filtered and washed with dry diethyl ether and dry hexane. The resulting solid was then vacuum dried for 2-3 h to afford the desired product. (108 mg, 0.77 mmol, 77% yield)
$^{1}$**H NMR** (400 MHz, $D_2O$) $\delta$ (ppm): $\delta$ 7.4 (s, 2H), 4.0 (s, 6H).
$^{13}$**C NMR** (101 MHz, $D_2O$) $\delta$ (ppm): $\delta$ 161.1, 158.3, 123.1, 36.7.
**HRMS (ESI+):** $m/z$ calculated for $C_6H_8N_2O_2Na$ $[M{+}Na]^+$: 163.0483; found: 163.0477, $m/z$ calculated for $C_5H_9N_2$ $[(M{-}CO_2){+}H]^+$: 97.0760; found: 97.0759.
**Direct inlet EIMS:** $m/z$ calculated for $C_5H_8N_2{}^+ = [M]^+$: 96.0, at 80 (±20) °C; found: $[M]^+$: 96.1.

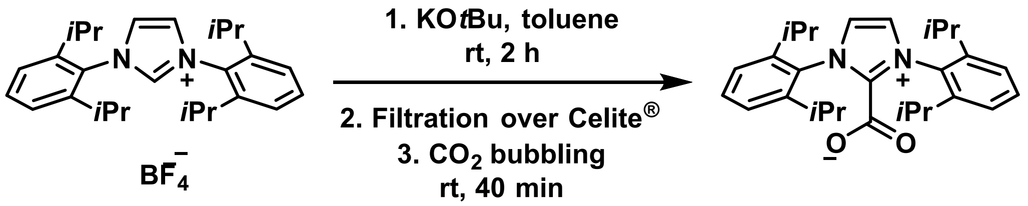


**1,3-bis(2,6-diisopropylphenyl)-1H-imidazol-3-ium-2-carboxylate (IPr-NHC · $CO_2$):** The compound is synthesized following a modified literature procedure.[20] 1,3-bis (2,6-diisopropylphenyl)-1H-imidazol-3-ium tetrafluoroborate (1.0 equiv., 0.5 mmol) and KO*t*Bu (1.0 equiv., 0.5 mmol) was taken in a flame dried Schlenk flask under argon. Dry toluene (10 mL) was added to the mixture and this was stirred at room temperature for 2 h. The suspension was then filtered under argon through a Celite® pad and carbon dioxide dried via conc. sulphuric acid was bubbled through the solution for 45 min. White solid precipitate was observed during this time, it was filtered and washed with dry hexane and dry diethyl ether. The resulting solid was vacuum dried to afford the desired product (170 mg, 0.39 mmol, 78% yield).

$^{1}$**H NMR** (400 MHz, $CD_2Cl_2$) $\delta$ (ppm): $\delta$ 7.5 (t, $J$ = 7.8 Hz, 2H), 7.3 (d, $J$ = 7.8 Hz, 4H), 7.2 (s, 2H), 2.5 (h, $J$ = 6.9 Hz, 4H), 1.3 (d, $J$ = 6.8 Hz, 12H), 1.2 (d, $J$ = 6.9 Hz, 12H).
$^{13}$**C NMR** (101 MHz, $CD_2Cl_2$) $\delta$ (ppm): $\delta$ 145.3, 132.9, 131.1, 124.6, 122.9, 29.6, 24.5, 23.6.
**HRMS (ESI+)**: $m/z$ calculated for $C_{27}H_{37}N_2{}^+$ $[(M{-}CO_2){+}H]^+$: 389.2951; found: 389.2948. **Direct inlet EIMS:** $m/z$ calculated for $C_{27}H_{36}N_2{}^+ = [M]^+$: 388.3, at 50 (±20) °C; found: $[M]^+$: 388.3.

## 4.3. Synthesis of NHO · $CO_2$ Adduct

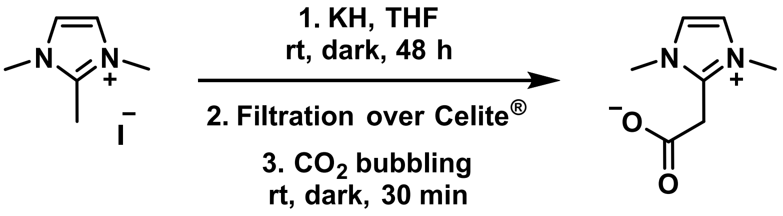


**2-(1,3-dimethyl-1H-imidazol-3-ium-2-yl) acetate (IMe-NHO · $CO_2$):** Following a literature procedure by Glorius and co-workers,[9] 1,2,3-trimethyl-1H-imidazol-3-ium iodide (1.0 equiv.) and KH (2.0 equiv.) were taken in a flame dried Schlenk flask. Dry THF was then added to same flask and it was stirred under argon at room temperature for 2 days. The flask was additionally covered with Al-foil to protect it from light. The suspension was then filtered under argon through a Celite® pad and carbon dioxide dried via conc. sulphuric acid was bubbled through the solution for 30 min maintaining a good light exclusion during this time. An off-white precipitate was observed during this time. This solid was filtered under ambient conditions and washed with dry hexane and dry diethyl ether. The final product was obtained after vacuum drying the obtained solid for 2-3 h (93 mg, 0.60 mmol, 60%).

$^{1}$**H NMR** (400 MHz, $D_2O$) $\delta$ (ppm): $\delta$ 7.4 (s, 2H), 4.0 (s, 2H), 3.8 (s, 6H).
$^{13}$**C NMR** (101 MHz, $D_2O$) $\delta$ (ppm): $\delta$ 171.6, 143.2, 122.2, 34.6, 32.1.
**HRMS (ESI+)**: $m/z$ calculated for $C_7H_{10}N_2O_2Na$ $[M{+}Na]^+$: 177.0640; found: 177.0632, $m/z$ calculated for $C_6H_{11}N_2$ $[(M{-}CO_2){+}H]^+$: 111.0917; found: 111.0914.
**Direct inlet EIMS:** $m/z$ calculated for $C_6H_{10}N_2{}^+ = [M]^+$: 110.1, at 40 (±20) °C; found: $[M]^+$: 110.1.

## 4.4. Synthesis of NHC · $HCO_3$ Adduct

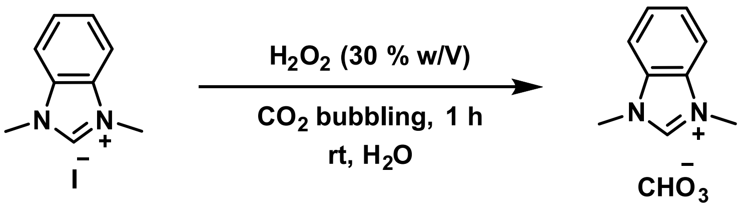


**1,3-dimethyl-1H-benzo[d]imidazol-3-ium bicarbonate (BIMe-NHC · $HCO_3$):** This compound was synthesized using a modified literature procedure by Crudden and coworkers.[21] 1,3-dimethylbenzimidazolium iodide (1.0 equiv., 1.9 mmol) was taken in a 50 ml round bottom flask and dissolved in 19 ml deionized water. The flask was equipped with a rubber septum and needles for addition of gaseous carbon dioxide and ventilation. $CO_2$ was bubbled through the solution for 1 min before addition of hydrogen peroxide 162 µl (30% w/v), in 0.4 ml of deionized water. $CO_2$ was purged through this mixture for additional 1 h before a purple precipitate was observed. The mixture was then filtered to remove iodine formed as purple solid and the clear solution was obtained. The water was removed using lyophilization to obtain a white solid as the desired product (255 mg, 1.22 mmol, 64% yield). We also performed the qualitative silver nitrate test to ensure complete removal of the iodine. 1 drop of the reaction aliquot was mixed with excess aqueous silver nitrate solution. A white precipitate of silver bicarbonate was observed that became colorless upon addition of 1M nitric acid.

$^{1}$**H NMR** (400 MHz, $D_2O$) $\delta$ (ppm): $\delta$ 7.9 (dt, $J = 6.8, 3.4$ Hz, 2H), 7.7 (dt, $J = 6.3, 3.4$ Hz, 2H), 4.1 (s, 6H). C2 proton is expected to exchange in $D_2O$.
$^{13}$**C NMR** (101 MHz, $D_2O$) $\delta$ (ppm): $\delta$ 160.3, 131.9, 126.7, 112.8, 32.7.
**HRMS (ESI+)**: $m/z$ calculated for $C_9H_{11}N_2^+$ $[M+H]^+$: 147.0917; found: 147.0916.
**Direct inlet EIMS:** $m/z$ calculated for $C_9H_{10}N_2^+ = [M]^+$: 146.1, at 50 ($\pm$20) °C; found: $[M]^+$: 146.1.

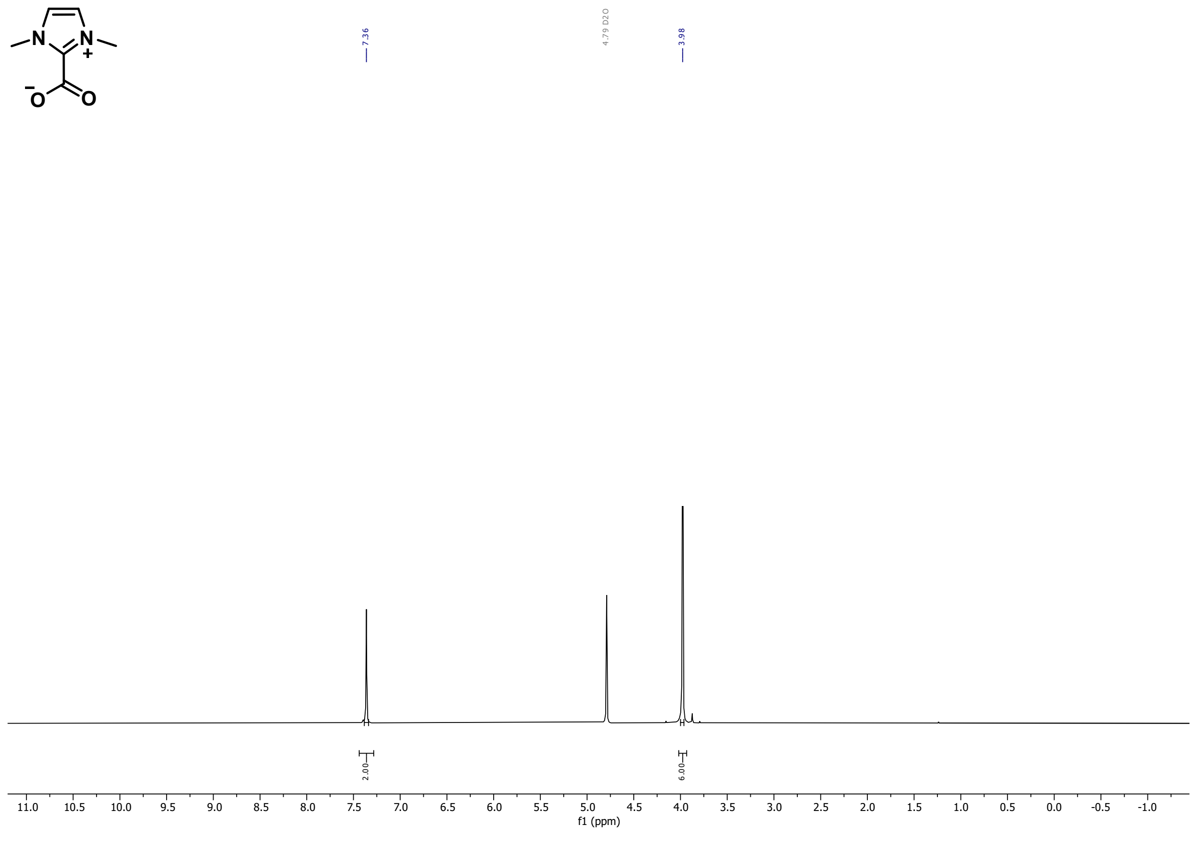

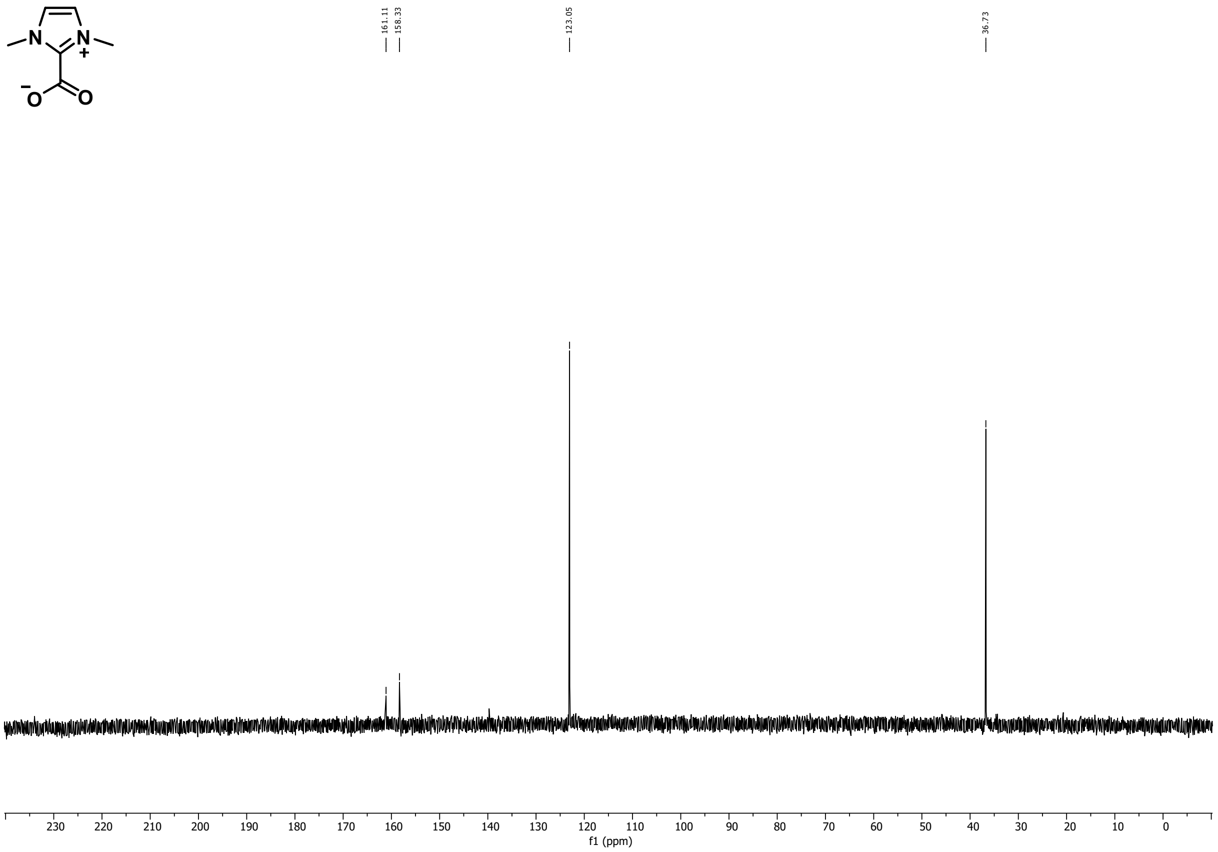
161.11
158.33
123.05
36.73
f1 (ppm)

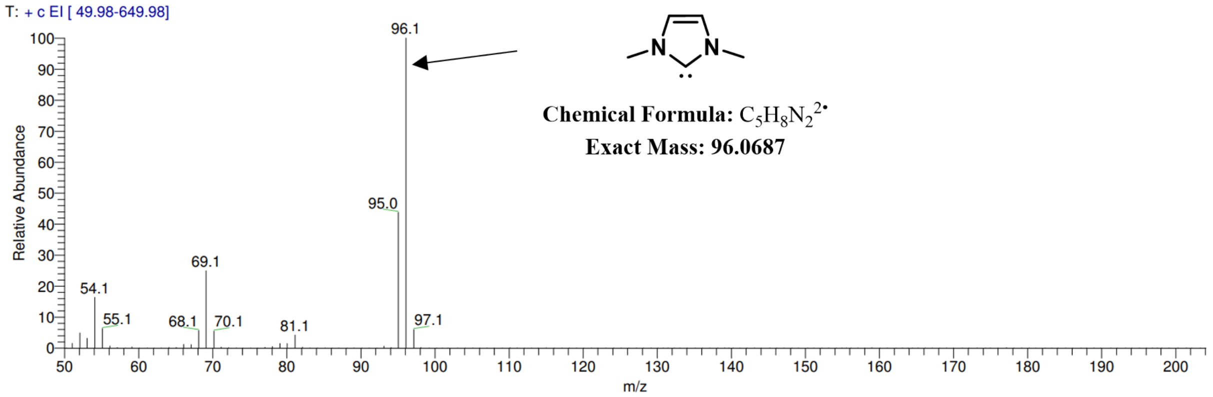
T: + c EI [ 49.98-649.98]
Relative Abundance
m/z
96.1
95.0
69.1
54.1
55.1
68.1
70.1
81.1
97.1
Chemical Formula: $C_5H_8N_2^{2•}$
Exact Mass: 96.0687

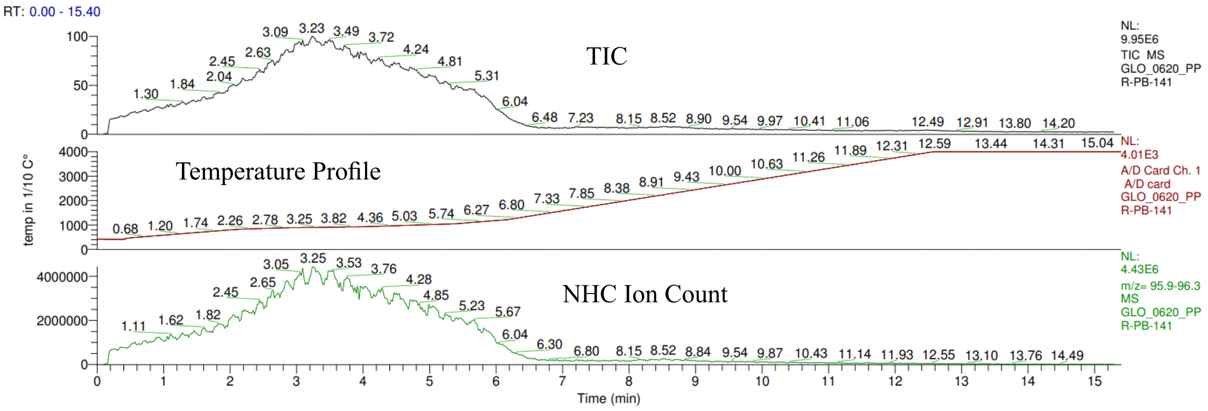
RT: 0.00 - 15.40
TIC
NL: 9.95E6 TIC MS GLO_0620_PP R-PB-141
Temperature Profile
temp in 1/10 C°
NL: 4.01E3 A/D Card Ch. 1 A/D card GLO_0620_PP R-PB-141
NHC Ion Count
NL: 4.43E6 m/z= 95.9-96.3 MS GLO_0620_PP R-PB-141
Time (min)

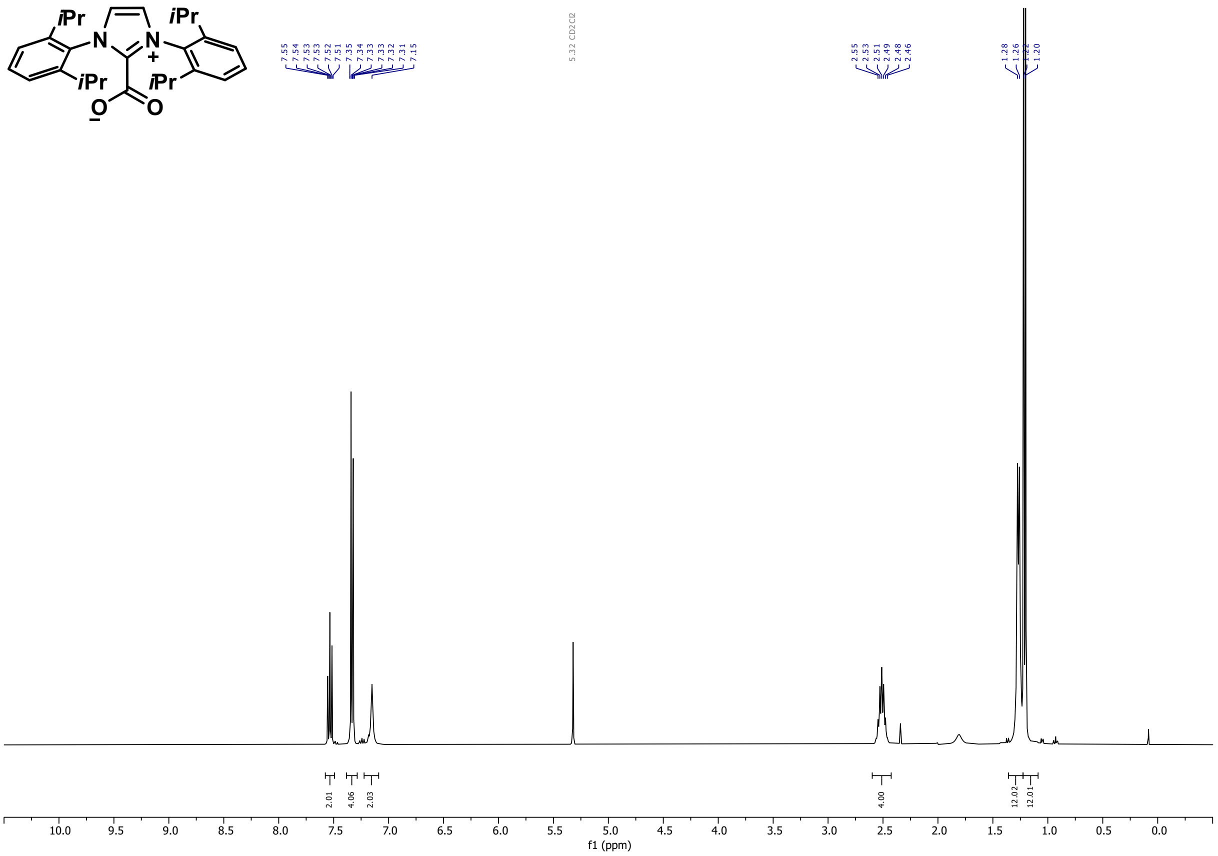

iPr
iPr
iPr
iPr
N
N
O
O
5.32 CD2Cl2
f1 (ppm)


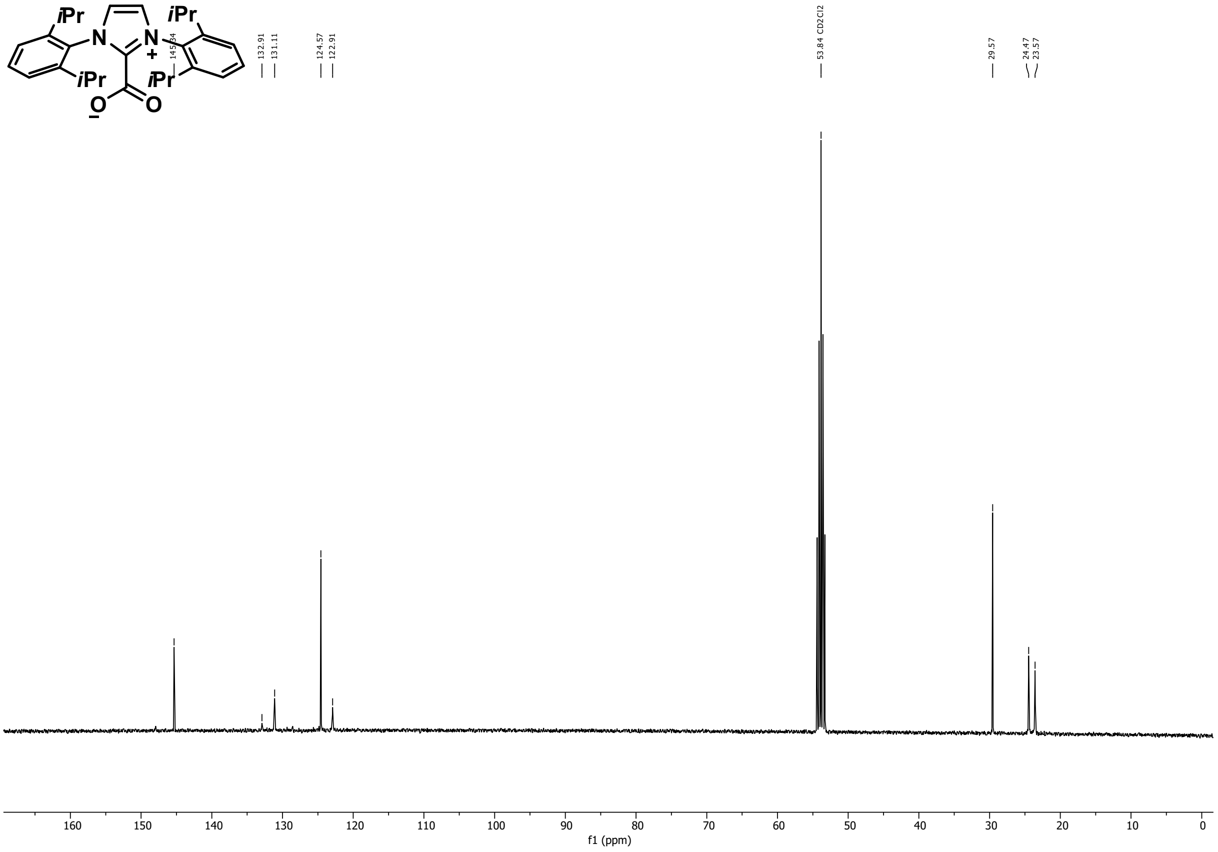

iPr
iPr
iPr
iPr
N
N
O
O
132.91
131.11
124.57
122.91
53.84 CD2Cl2
29.57
24.47
23.57
f1 (ppm)

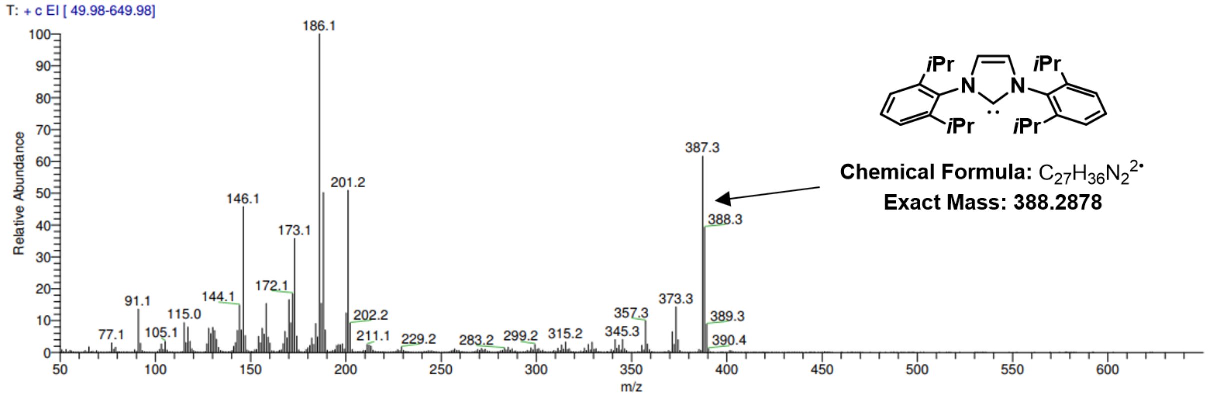
T: + c EI [ 49.98-649.98]
Relative Abundance
m/z
186.1
387.3
201.2
146.1
173.1
388.3
172.1
144.1
91.1
115.0
77.1
105.1
202.2
211.1
229.2
283.2
299.2
315.2
345.3
357.3
373.3
389.3
390.4
iPr
iPr
iPr
iPr
N
N
Chemical Formula: $C_{27}H_{36}N_2^{2\bullet}$
Exact Mass: 388.2878

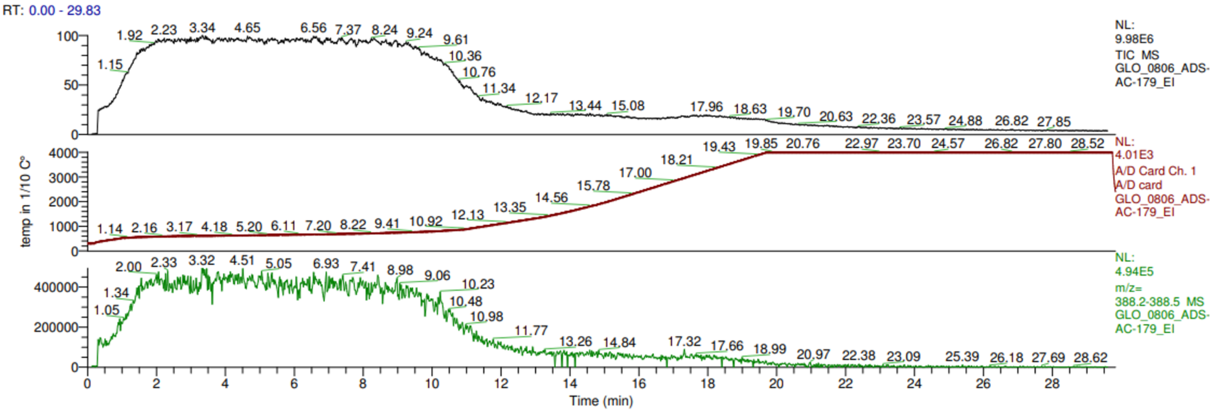
RT: 0.00 - 29.83
NL: 9.98E6
TIC MS
GLO_0806_ADS-AC-179_EI
NL: 4.01E3
A/D Card Ch. 1
A/D card
GLO_0806_ADS-AC-179_EI
NL: 4.94E5
m/z= 388.2-388.5 MS
GLO_0806_ADS-AC-179_EI
temp in 1/10 C°
Time (min)

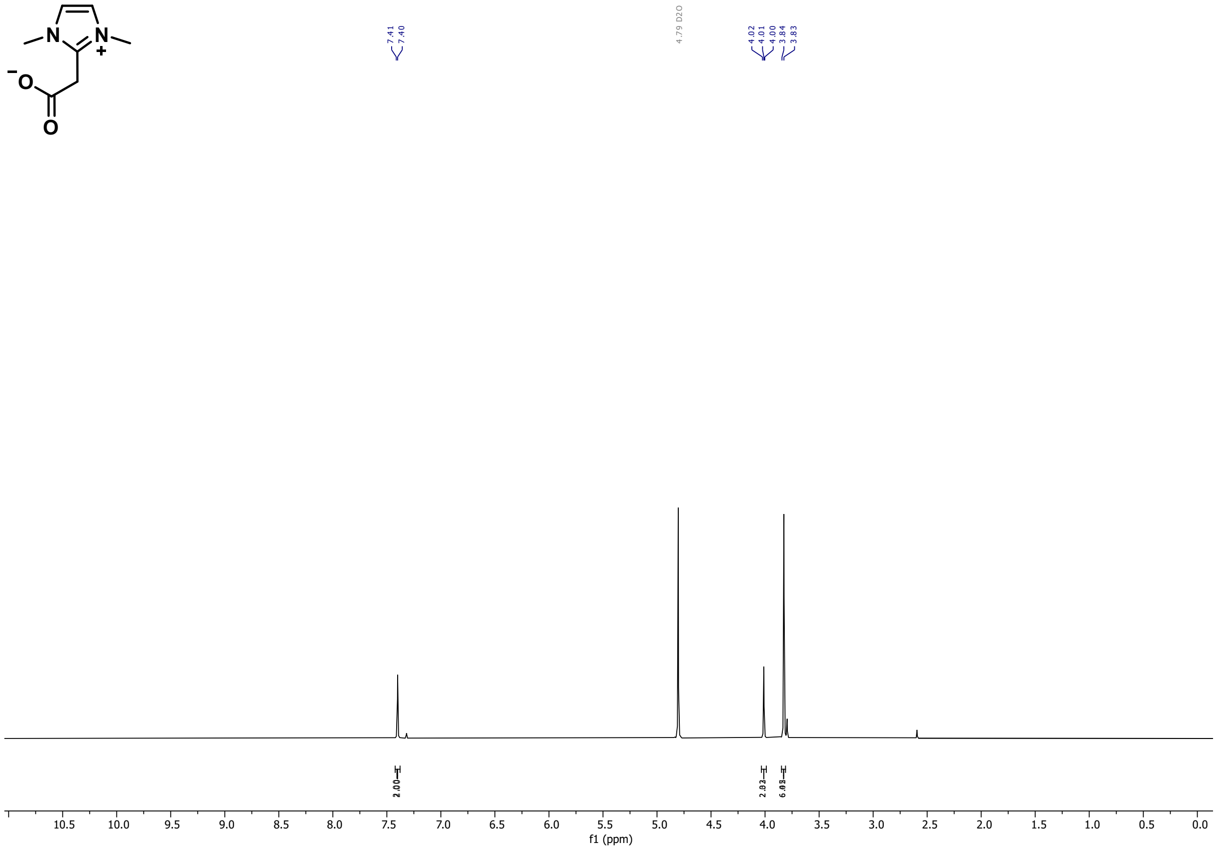
N
N
+
O
O
-
7.41
7.40
4.79 D2O
4.02
4.01
4.00
3.84
3.83
2.00
2.03
6.03
f1 (ppm)

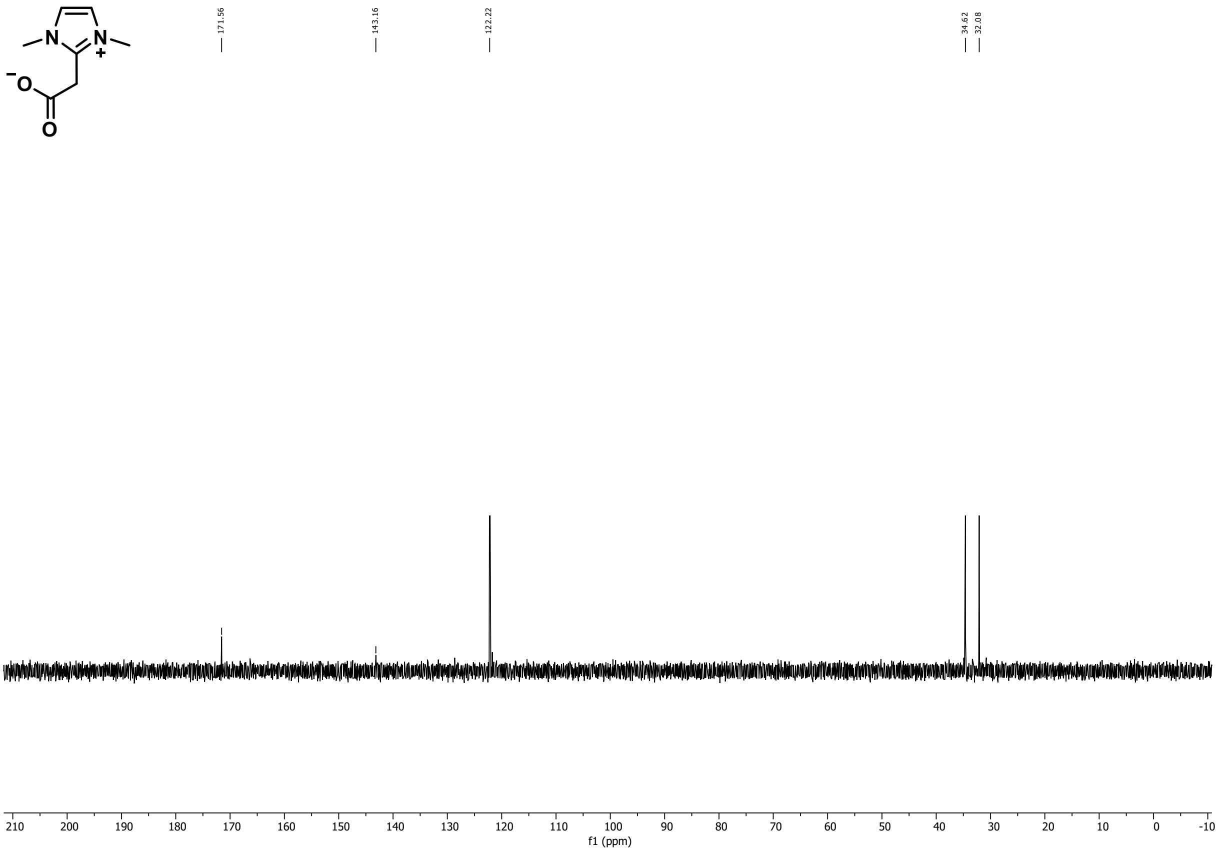
171.56
143.16
122.22
34.62
32.08
f1 (ppm)

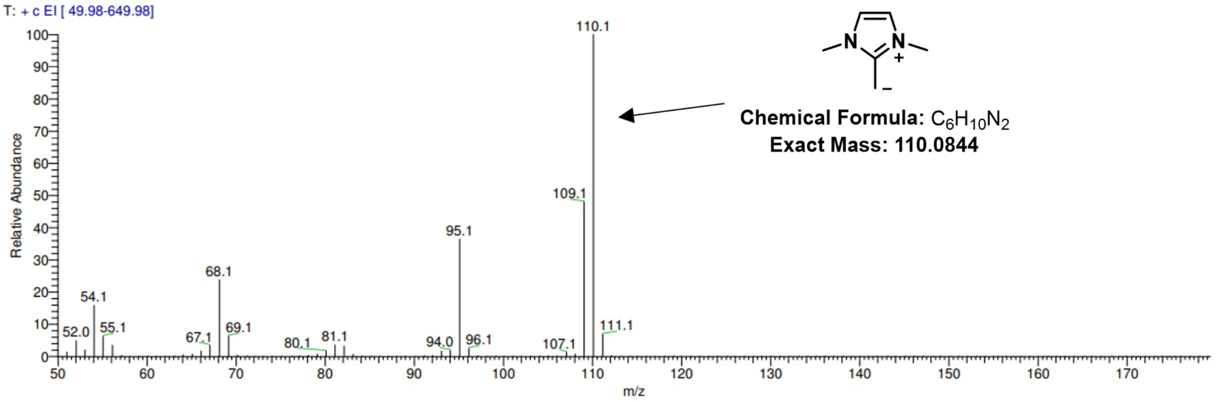
T: + c EI [ 49.98-649.98]
Relative Abundance
m/z
110.1
109.1
95.1
68.1
54.1
Chemical Formula: $C_6H_{10}N_2$
Exact Mass: 110.0844

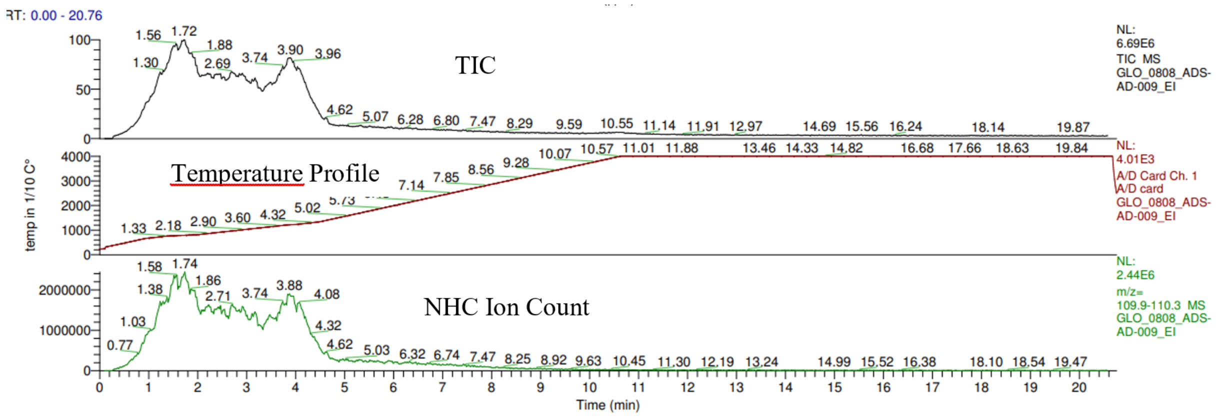
RT: 0.00 - 20.76
TIC
Temperature Profile
NHC Ion Count
temp in 1/10 C°
Time (min)
NL: 6.69E6 TIC MS GLO_0808_ADS-AD-009_EI
NL: 4.01E3 A/D Card Ch. 1 A/D card GLO_0808_ADS-AD-009_EI
NL: 2.44E6 m/z= 109.9-110.3 MS GLO_0808_ADS-AD-009_EI

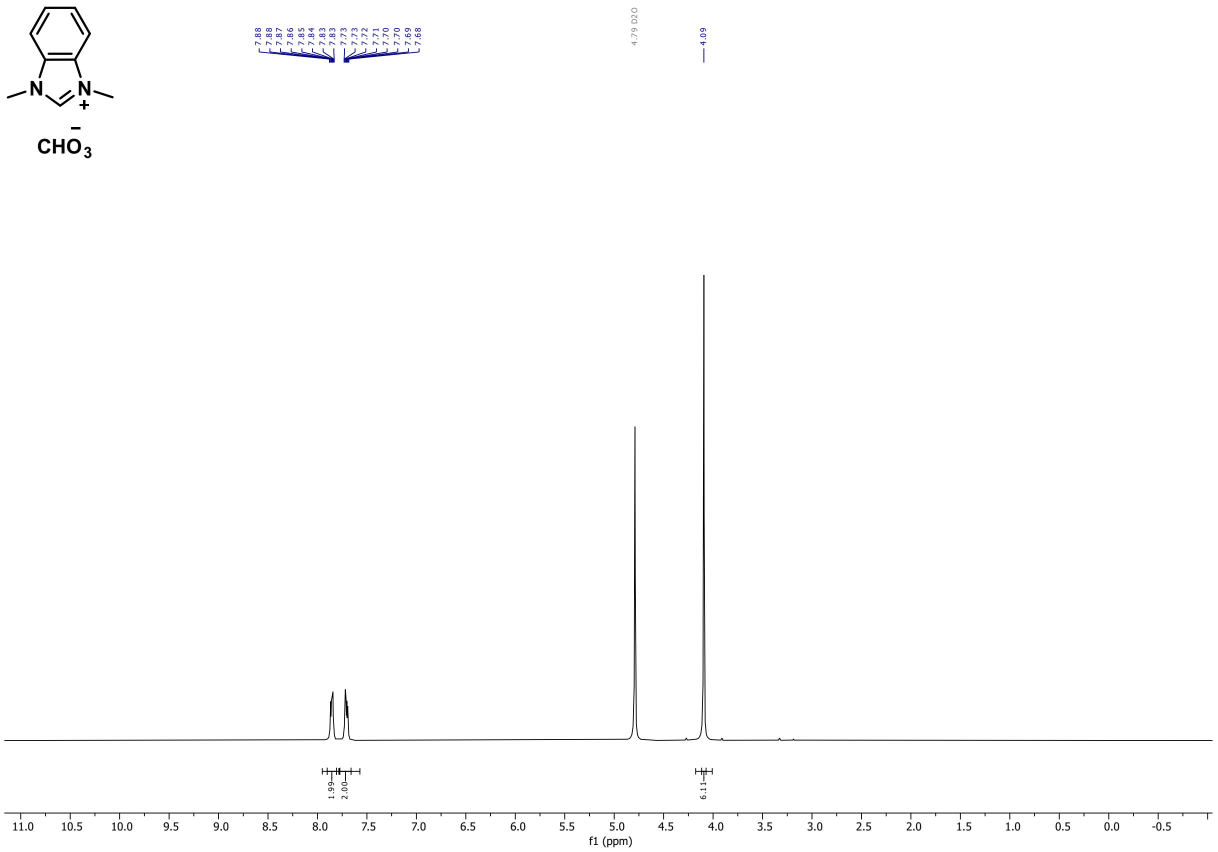
CHO3
7.88
7.88
7.87
7.86
7.85
7.84
7.83
7.83
7.73
7.73
7.72
7.71
7.70
7.70
7.69
7.68
4.79 D2O
4.09
1.99
2.00
6.11
f1 (ppm)

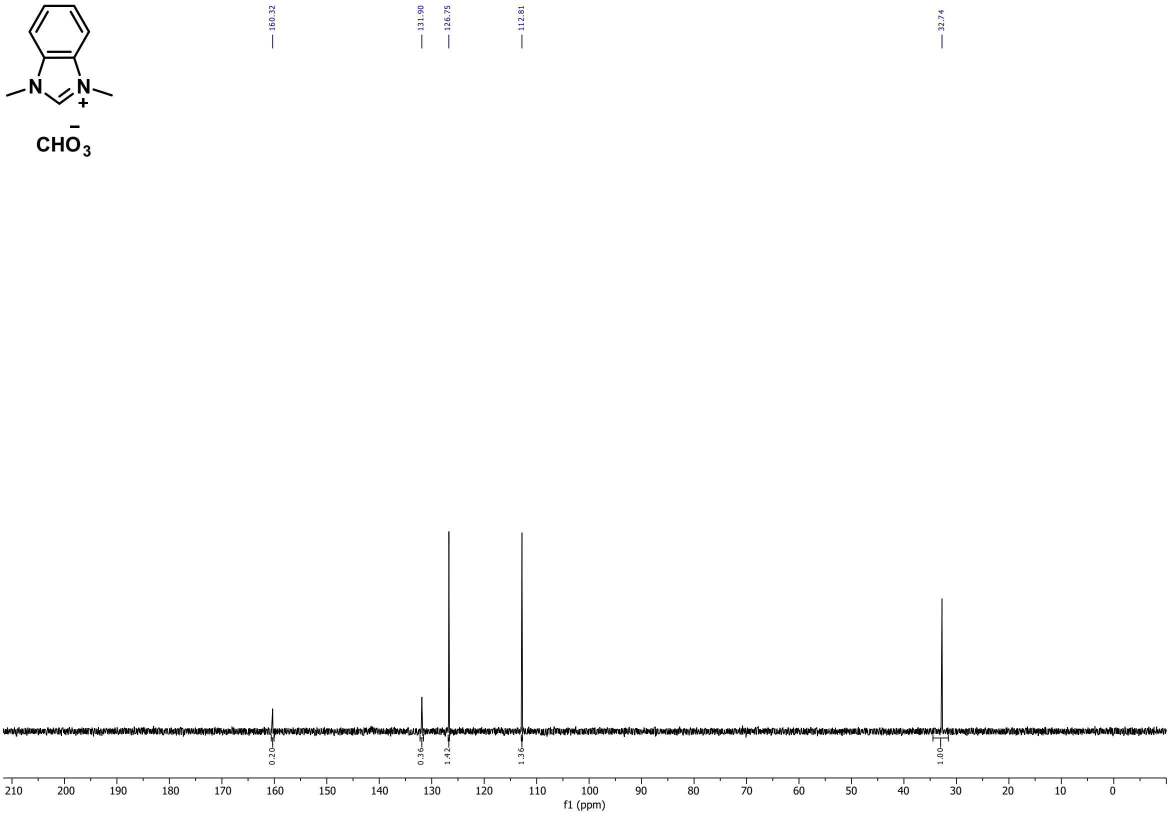
CHO3
160.32
131.90
126.75
112.81
32.74
0.20
0.36
1.42
1.36
1.00
f1 (ppm)

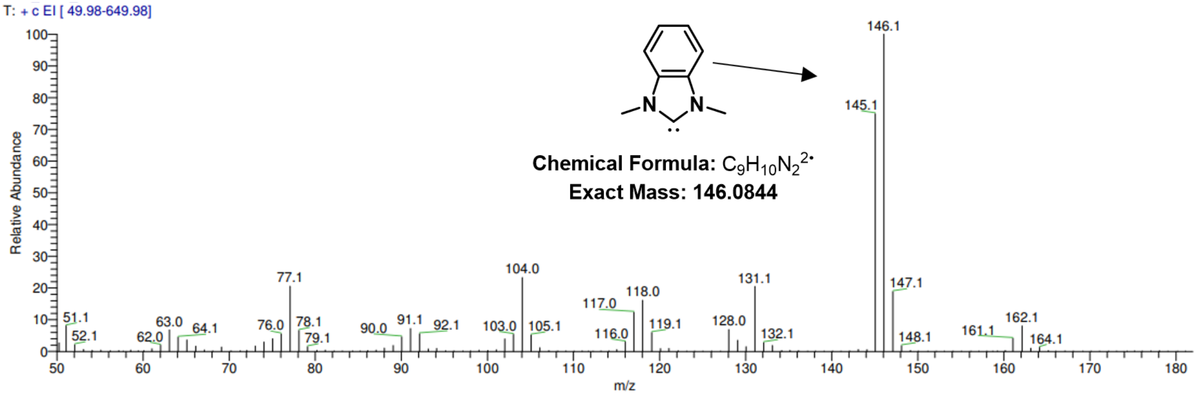
T: + c EI [ 49.98-649.98]
Relative Abundance
m/z
146.1
145.1
147.1
77.1
104.0
118.0
117.0
131.1
162.1
Chemical Formula: $C_9H_{10}N_2^{2\bullet}$
Exact Mass: 146.0844

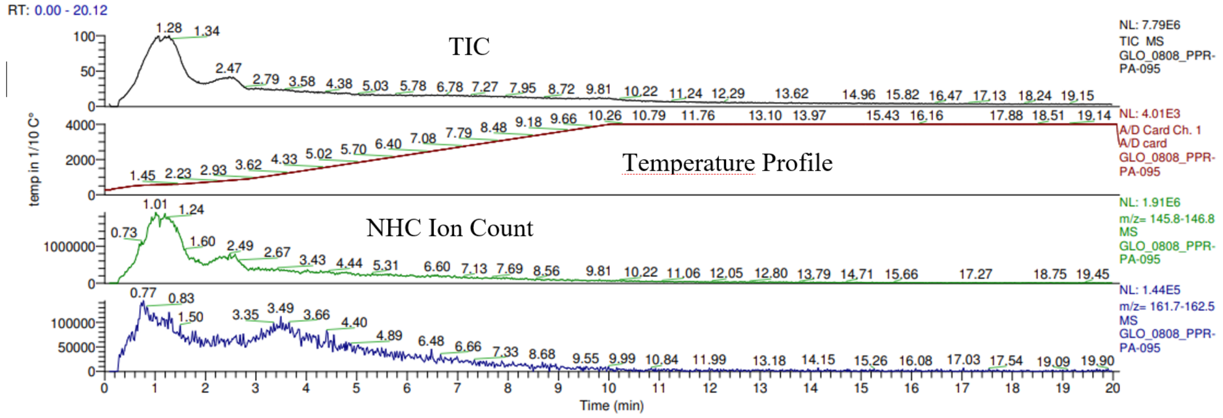
RT: 0.00 - 20.12
TIC
Temperature Profile
NHC Ion Count
temp in 1/10 C°
Time (min)
NL: 7.79E6
TIC MS
GLO_0808_PPR-PA-095
NL: 4.01E3
A/D Card Ch. 1
A/D card
GLO_0808_PPR-PA-095
NL: 1.91E6
m/z= 145.8-146.8
MS
GLO_0808_PPR-PA-095
NL: 1.44E5
m/z= 161.7-162.5
MS
GLO_0808_PPR-PA-095

# 5. The GaAs(110) Surface

Like most III-V compound semiconductors (e.g. InAs, GaSb, and GaP), GaAs crystallizes in the cubic Zincblende structure. For these crystals, the non-polar (110) surface, which is used here, is the perferred cleavage plane. The structure model of this surface, which is characterized by a relaxation with $(1 \times 1)$ periodicity,[22–26] is shown in Figure S1(A). The relaxation leads to a displacement of the surface Ga and As atoms with respect to their bulk positions. The As surface atoms relax outward relative to their bulk position, while the Ga atoms slightly relax inwards. This is accompanied by a charge transfer from the Ga atoms to the As atoms and results in a structure, where the Ga atoms approach a planar threefold coordination with filled $sp^2$-like and empty $p_z$-like orbitals, while the As atoms adopt a three-dimensional tetrahedral $sp^3$-like configuration with a doubly occupied dangling bond. The GaAs(110) surface thus offers two chemically inequivalent sites for molecule adsorption, at the As atoms (filled dangling bonds) and at the Ga atoms (empty dangling bonds). Optical transitions between these surface localized electronic states appear around 2.6 eV, providing a spectroscopic signature of the surface electronic band structure of the relaxed surface.[27]

The GaAs(110) surface is characterized by a rectangular unit cell (red boxes in Figure S1) with a length in [001] direction of $a_{[001]} = a_0 = 0.565$ nm corresponding to the cubic GaAs lattice constant $a_0$ and a length of $a_{[1\bar{1}0]} = a_0/\sqrt{2} = 0.400$ nm in $[1\bar{1}0]$ direction. The rectangular $(1 \times 1)$ unit cell of the relaxed GaAs(110) surface is also visible in LEED measurements as shown in Figure S2.

Due to the presence of an empty $p_z$-like orbital at the Ga atoms and a fully occupied orbital at the As atoms, STM selectively images the Ga atoms with positive sample bias (empty states), while at negative sample bias (filled states) the As atoms are imaged[28]. Examples of such filled states STM images showing the As atoms are presented in Figure S1(B,C). The cleavage surface exhibits a very low defect density, as is evident from the overview image shown in (B). Thus, defects do not play a significant role for the adsorption and assembly behavior of the NHC and NHO molecules.

The clean GaAs surface was furthermore characterized using XPS. Spectra of the Ga $3d$ and As $3d$ core levels are presented in Figure S3. They consist of spin-orbit split doublets and can be decomposed in one bulk and one surface component each, in accordance with literature.[29,30]

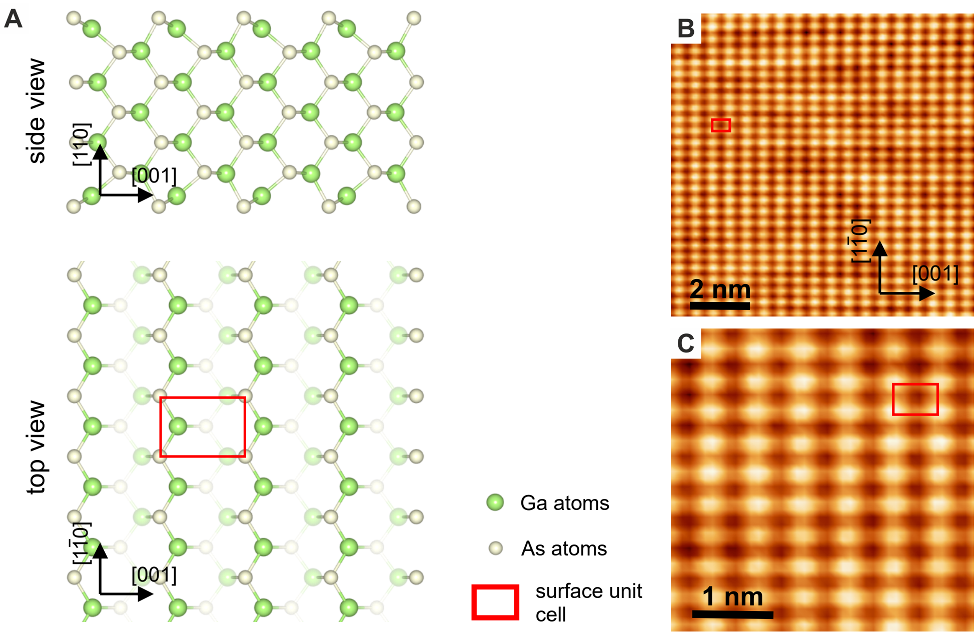


**Figure S1.** The GaAs(110) surface. (A) Structure model of the relaxed GaAs(110) surface in side view and top view. (B,C) Filled states STM images of the surface (sample voltage $V_T = -2.0$ V; tunneling current $I_T = 50$ pA). The surface unit cell is indicated in red.

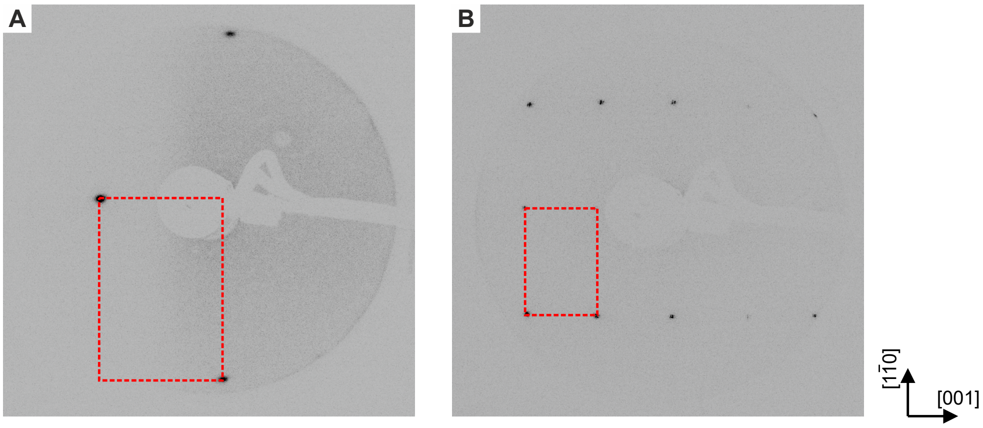


**Figure S2.** LEED diffraction patterns of the clean GaAs(110) surface obtained at (A) $E_{kin} = 20$ eV and (b) $E_{kin} = 60$ eV. The surface unit cell is indicated in red.

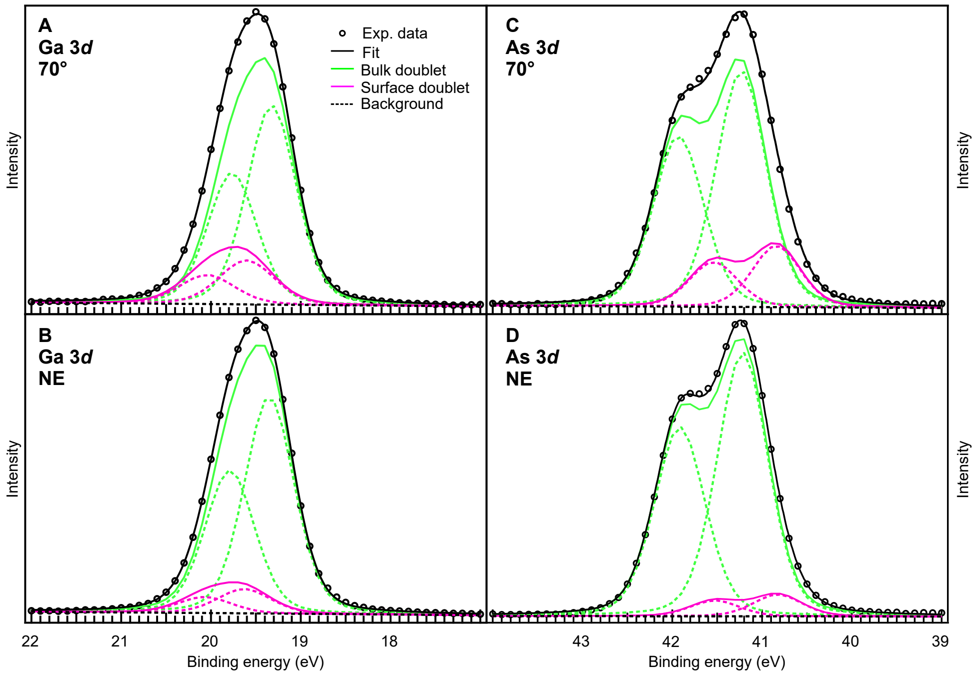


**Figure S3.** XPS results for (A,B) the Ga $3d$ and (C,D) the As $3d$ core levels of the clean GaAs(110) surface. To vary the surface sensitivity, the spectra were obtained under normal emission (NE) and under an angle of 70° with respect to the surface normal.

# 6. Adsorption Geometries from DFT

**Table S1.** Total adsorption energy and the vdW and internal strain component for each molecule in its isolated adsorption geometry. The strain is defined as the energy difference between the molecule in its gas-phase geometry and in its deformed or 'peeled-off' adsorption geometry.

| Molecule | $E^{ads}_{total}$ (eV) | $E^{ads}_{vdW}$ (eV) | $E^{ads}_{strain}$ (eV) |
|---|---|---|---|
| IMe-NHC | −2.07 | −0.71 | +0.12 |
| BIMe-NHC | −1.99 | −0.77 | +0.11 |
| IMe-NHO | −1.91 | −0.57 | +0.36 |
| IPr-NHC(1) | −1.66 | −1.95 | +0.52 |
| IPr-NHC(2) | −1.60 | −1.79 | +0.08 |

## 6.1. BIMe-NHC, IMe-NHC, and IMe-NHO

For all molecules with methyl sidegroups, DFT finds an adsorption at the Ga surface atoms with a covalent bond formation and an orientation of the central heterocyclic ring along the Ga-As rows of the GaAs(110) surface, i.e. the [1$\bar{1}$0] direction. The DFT geometries for isolated molecules are shown in Figure S4 and their adsorption energies are reported in Table S1. The following Figures S5–S9 present the geometries for molecule pairs and chains. In addition to the isolated monomer geometry, a 'separate' geometry was also investigated in which two molecules are as far away from each other as possible within the same simulation cell. The determined tilt angles are listed in Table S2.

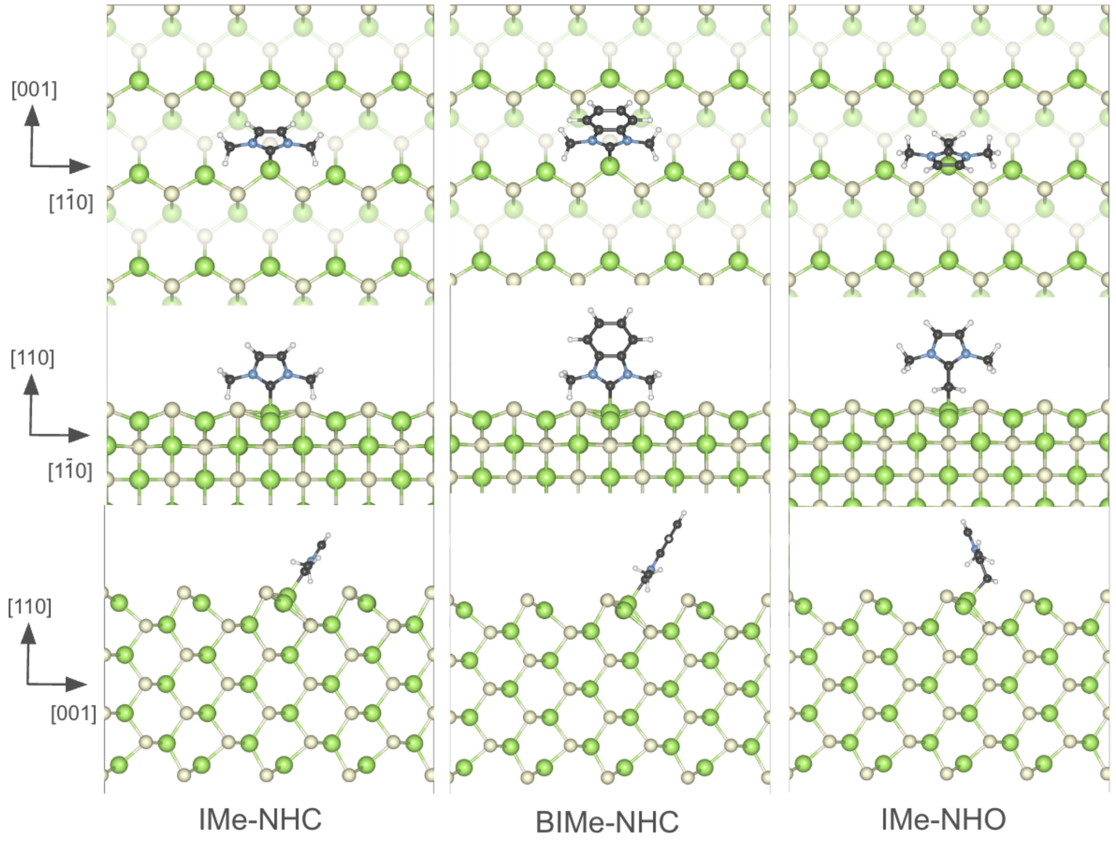


**Figure S4.** Geometries for isolated adsorption of IMe-NHC, BIMe-NHC, and IMe-NHO on GaAs(110). The same crystallographic directions are used in subsequent figures.

**Table S2.** Tilt angles of the heterocyclic ring with respect to the surface normal for the geometries shown in Figures S4–S10. $\theta_1$ and $\theta_2$ are the tilt angles for each molecule in a pair geometry. The difference $\Delta\theta$ quantifies the coupling between molecules.

| Molecule | Geometry | $\theta_1$ | $\theta_2$ | $\Delta\theta$ |
|---|---|---|---|---|
| IMe-NHC | isolated | 32.7° | | |
| | rows | 32.6° | 32.3° | 0.3° |
| IMe-NHO | isolated | −23.8° | | |
| | rows | −24.3° | −24.1° | −0.2° |
| BIMe-NHC | isolated | 30.3° | | |
| | separate | 30.3° | 30.3° | 0.0° |
| | diagonal | 27.3° | 41.6° | 14.3° |
| | rows | 24.8° | 44.8° | 20.0° |
| | chains | 44.6° | | |
| IPr-NHC | geometry 1 | 8.2° | | |
| IPr-NHC | geometry 2 | 4.2° | | |

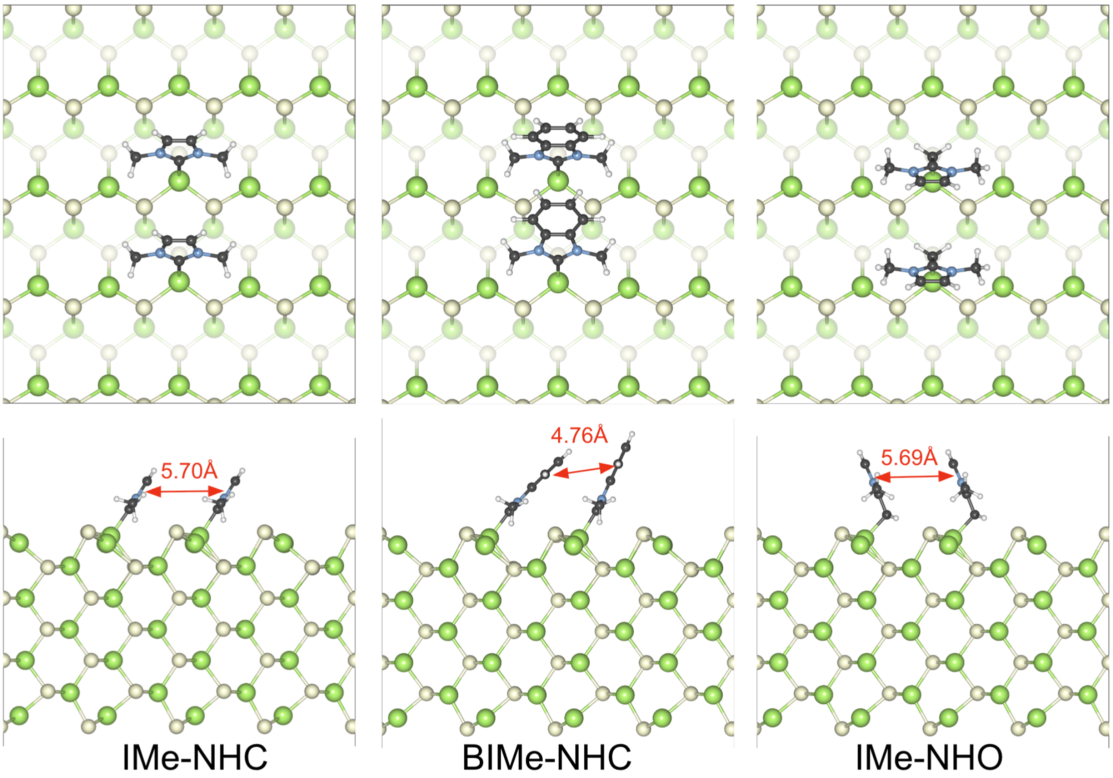


**Figure S5.** Geometries for pairwise adsorption of IMe-NHC, BIMe-NHC, and IMe-NHO on GaAs(110) in rows. $\pi$–$\pi$ distances are indicated.

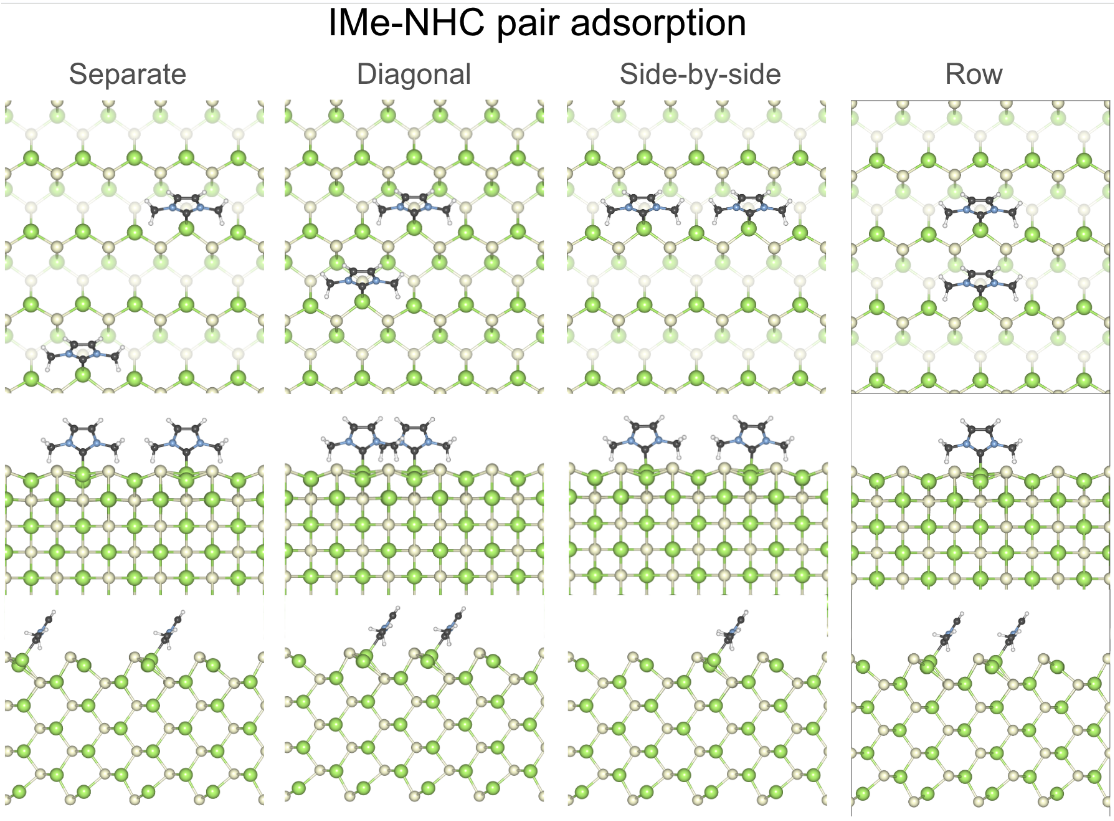


**Figure S6.** Geometries for pairwise adsorption of IMe-NHC on GaAs(110).

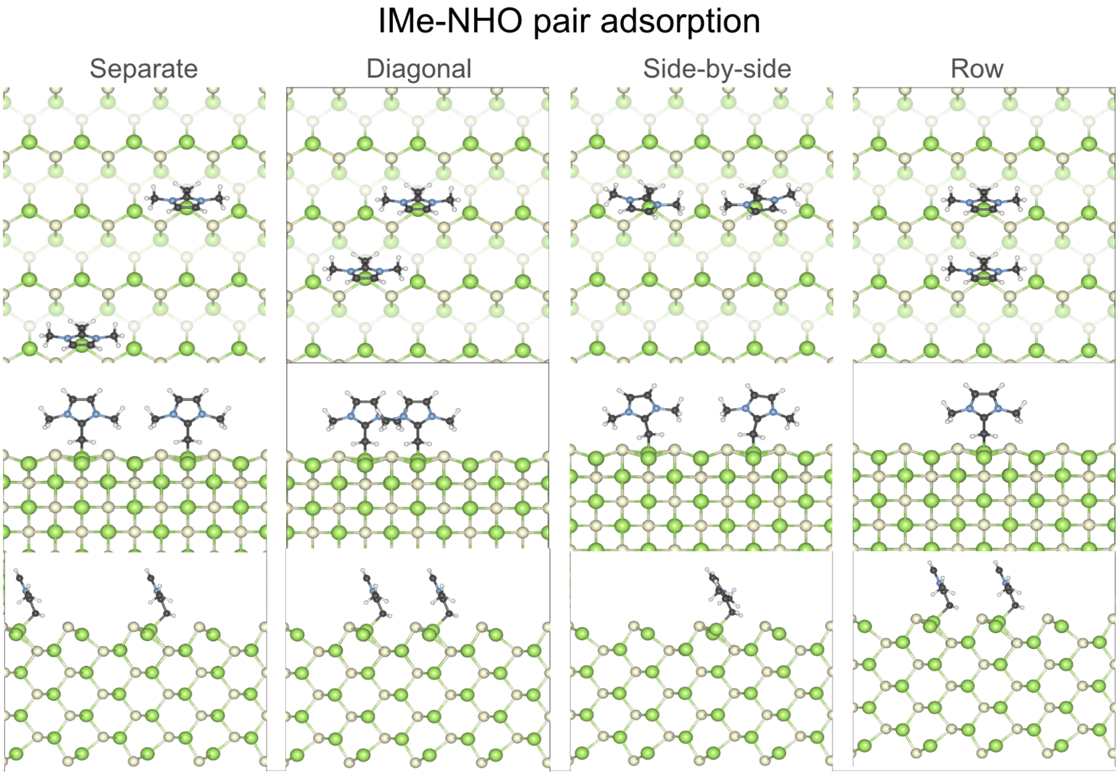


**Figure S7.** Geometries for pairwise adsorption of IMe-NHO on GaAs(110).

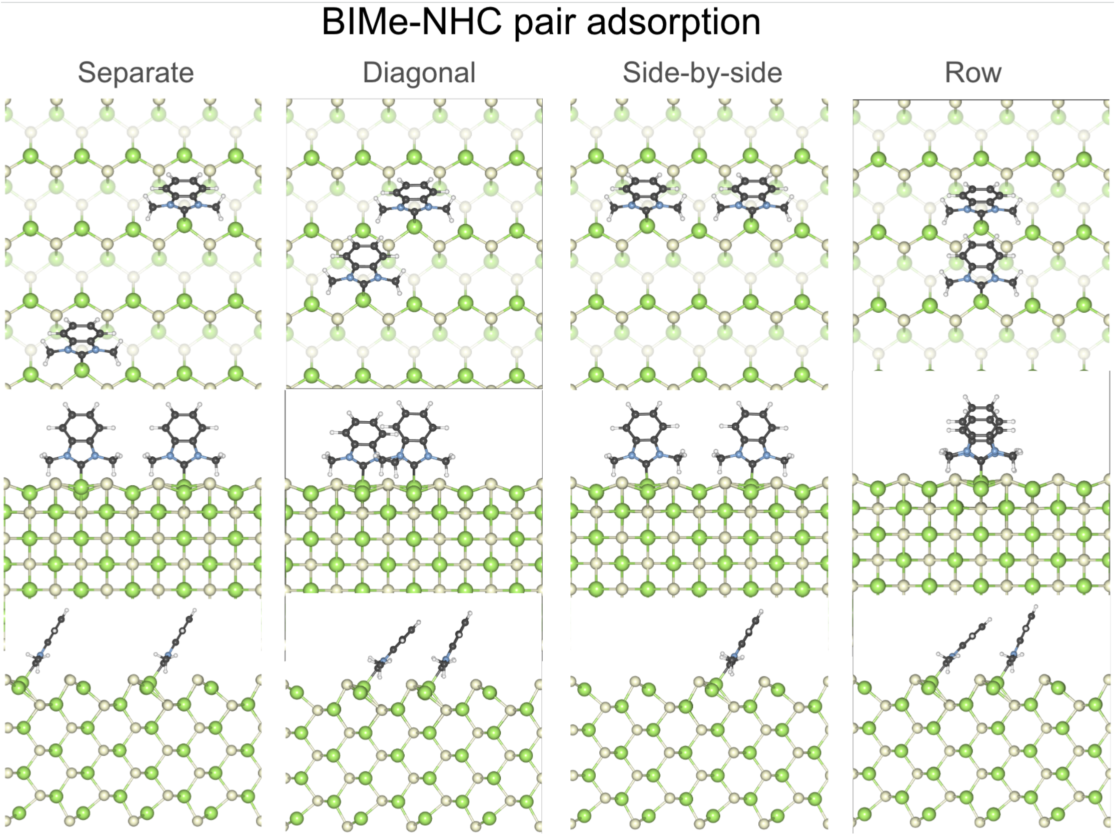


**Figure S8.** Geometries for pairwise adsorption of BIMe-NHC on GaAs(110).

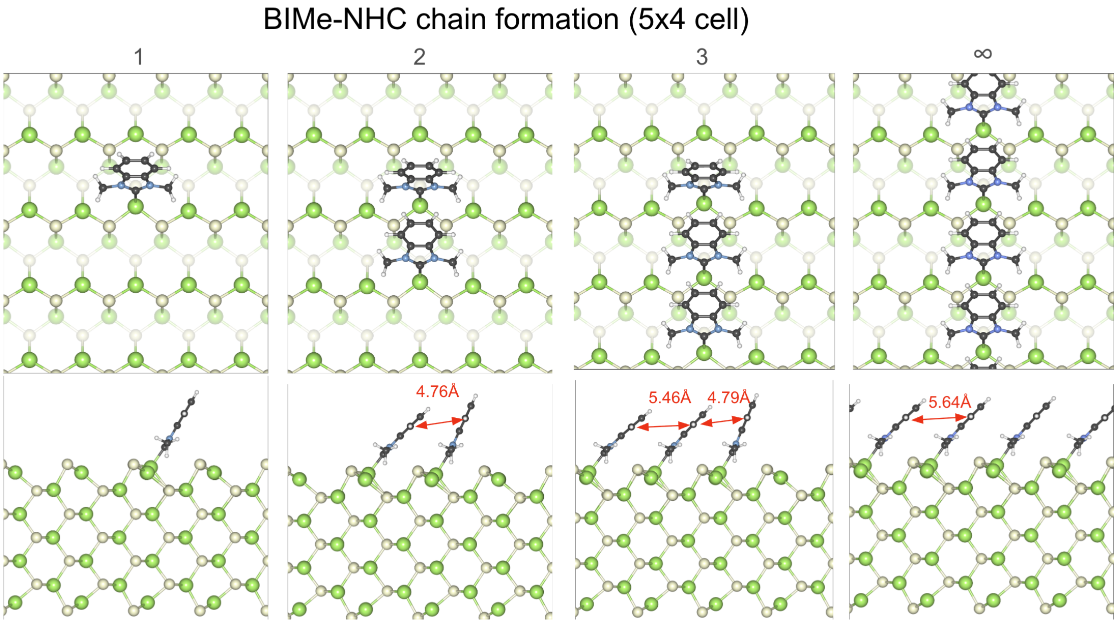


**Figure S9.** Geometries for short ($n = 1, 2, 3$) and infinitely long chains of BIMe-NHC along $[1\bar{1}0]$ on GaAs(110). $\pi$–$\pi$ distances are indicated.

## 6.2. IPr-NHC

Our DFT calculations for isolated IPr-NHC molecules reveal large differences as compared to the other molecules with methyl sidegroups (see Figure S10). An orientation with the central heterocyclic ring being oriented along the Ga-As rows (i.e. along the $[1\bar{1}0]$ direction), which is the most favorable geometry for the other molecules, is not possible. Instead, two geometries with almost identical adsorption energies are found.

In geometry 1, which is found to be energetically slightly more favorable, the central ring is aligned along the [001] direction. Here, chemical bond formation is possible and the Ga-C distance is rather small ($d_{\mathrm{Ga-C}} = 2.3$ Å). In geometry 2, the molecule slightly rotates and adsorbs in a way with two isopropyl groups locked in the $[1\bar{1}0]$ trenches of the GaAs surface. Thereby, the molecule remains physisorbed and relatively far away from the surface, with only weak interaction between the carbene C atom and the Ga surface atom (Ga-C distance $d_{\mathrm{Ga-C}} = 3.89$ Å).

Nevertheless, the binding for both situations is dominated by a large van der Waals (vdW) interaction: of the overall adsorption energy of $E_{\mathrm{ads}} = -1.66$ eV ($E_{\mathrm{ads}} = -1.60$ eV) for geometry 1 (2), the vdW interaction accounts for $-1.95$ eV ($-1.79$ eV). This shows that a strong repulsive interaction must also be present that originates from the stress in the sidegroups. In order to draw closer to the surface and thus maximize both attractive Ga-C chemical bond and vdW interactions, the IPr molecule sidegroups must flatten, resulting in a considerable internal strain penalty. Competition among these various interaction components (surface relaxation and other contributions are implicit) determines the final geometry and adsorption energy.

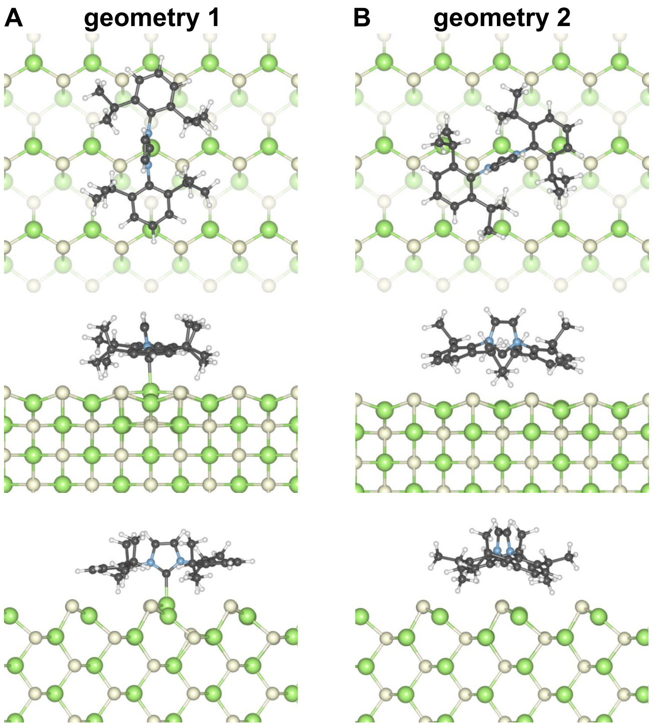


**Figure S10.** Geometries for IPr-NHC on GaAs(110).

# 7. Charge Density Analysis from DFT

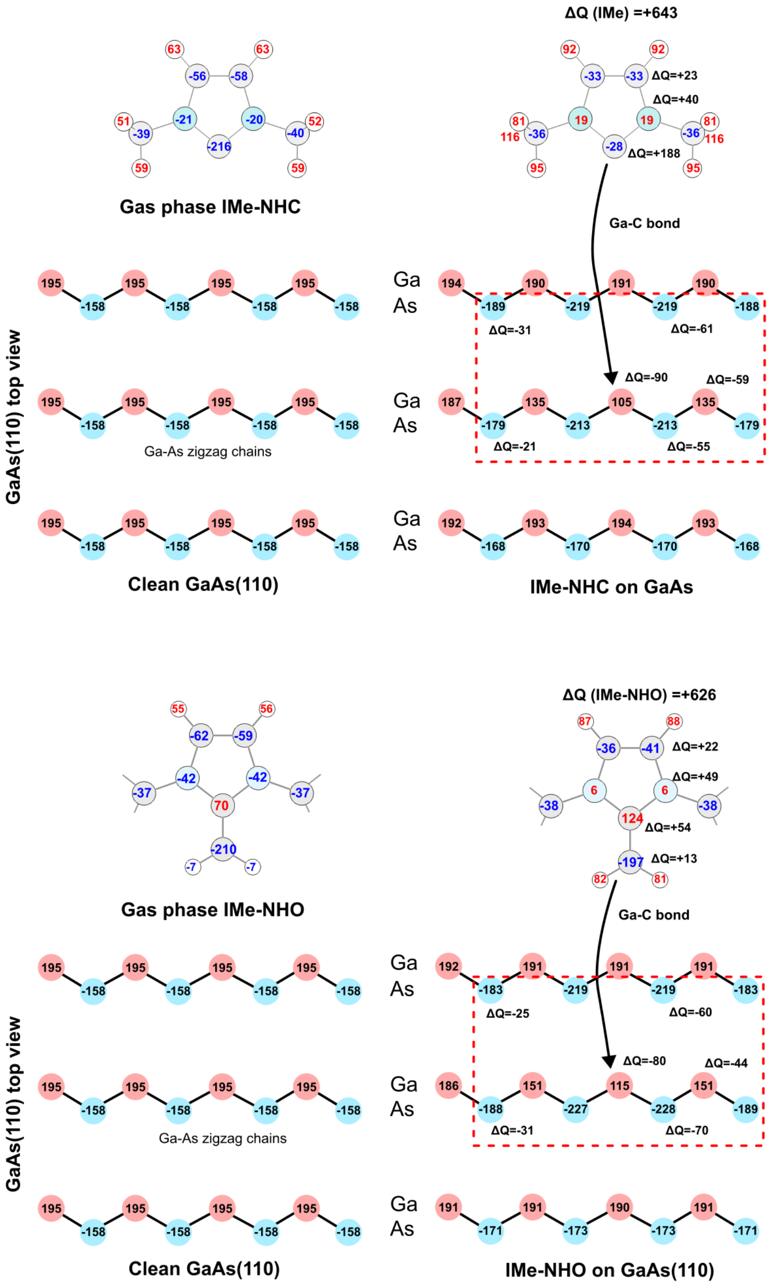


**Figure S11.** Voronoi deformation density (VDD) analysis for IMe-NHC and IMe-NHO adsorption on GaAs(110), in terms of atomic charges and charge differences $\Delta Q$ (in millielectrons). Part of the GaAs(110) surface around the adsorption site is shown as a top view (topmost Ga-As layer only). All geometries are shown schematically. The dashed rectangle indicates the region around the binding site in which the charge transfer is greatest.

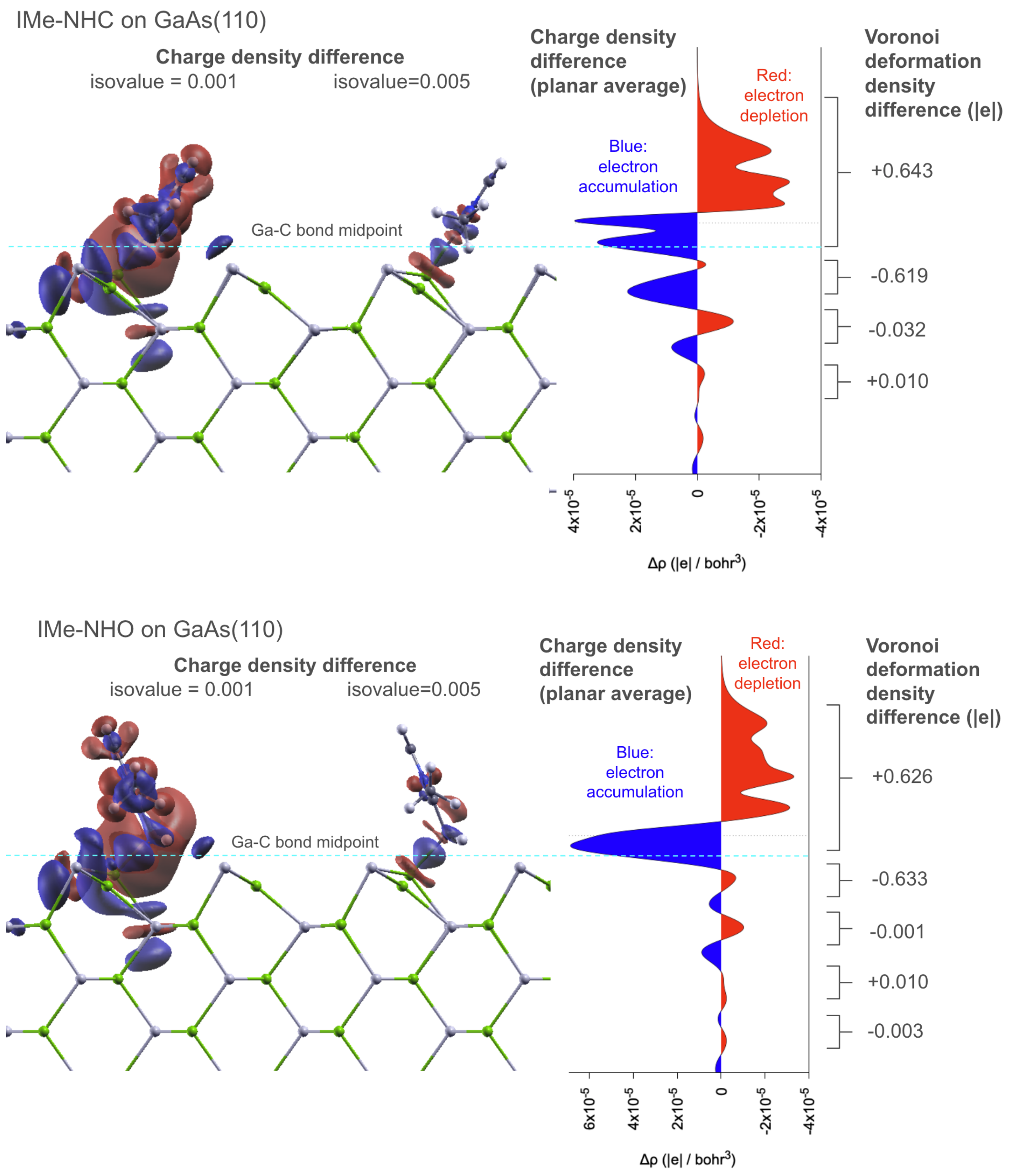


**Figure S12.** Left: Charge density difference (CDD) isosurfaces and planar-averaged CDD $\Delta\rho = -(\rho_{\mathrm{NHC/GaAs(110)}} - \rho_{\mathrm{NHC}} - \rho_{\mathrm{GaAs(110)}})$, for IMe-NHC (top) and IMe-NHO (bottom) adsorption on GaAs(110). Right: Voronoi deformation density charge differences ($\Delta Q$, in electrons) summed over the atoms in the indicated regions, with respect to the gas phase molecule and pristine surface. The CDD and VDD have been defined here to give consistent signs, i.e. positive values indicate electron depletion (shown in red).

# 8. Determination of the Adsorption Site from STM

## 8.1. BIMe-NHC, IMe-NHC, and IMe-NHO

As described in section 5, STM selectively images either the Ga or the As atoms of the GaAs(110) surface depending on the polarity of the tunneling voltage. In filled states STM images (negative sample voltage $V_T$), the As surface atoms are imaged, while empty states STM images (positive $V_T$) show the locations of the Ga surface atoms. When atomic resolution is achieved, this allows us to determine the adsorption site, as demonstrated in the main text for BIMe-NHC.

An analogous analysis is shown in Figure S13 for IMe-NHC on the basis of both filled (A-C) and empty states (D-F) STM data. In the filled states image, the maximum of the molecule appears atop a substrate minimum, while in the empty states image the maximum of the molecule appears above a maximum of the substrate. This clearly proves the adsorption at the Ga atoms of the GaAs(110) surface as also found in the DFT calculations (cf. Figure S4).

In Figure S14, a corresponding analysis is presented for IMe-NHO, again giving experimental proof for the DFT results. Thus, the STM data for all molecules with methyl sidegroups show a binding to the Ga atoms, in agreement with the DFT calculations.

## 8.2. IPr-NHC

High resolution STM data for low IPr-NHC coverages on the GaAs(110) surface are presented in Figure S15. In such images of low IPr-NHC coverages, molecules appearing with two different heights are observed, as indicated by the blue and yellow circles. These two appearances are assigned to the two geometries 1 and 2 (see Figure S10), exhibiting similar adsorption energies as found in DFT. The lower appearing molecules (blue) are assigned to geometry 1, in which the molecules are chemisorbed and relatively close to the surface, in agreement with the considerably smaller apparent height found in profile 1 as compared to profile 2 for the higher appearing molecules (Figure S15(D,E)). Moreover, the flatter top observed in Figure S15(D) mimics the flattening of the molecule due to the covalent-bond formation. For both geometries the center of the molecule is located near a Ga surface atom, in agreement with (C) showing the positions of the Ga chains as white lines.

For higher IPr-NHC coverages, the lower appearing molecules become dominant. In Figure 2(K) only few brighter appearing IPr-NHC molecules are present, and in the monolayer (Figure 2(L)) almost all molecules appear with similar (lower) height. This shows that geometry 1, being the lowest energy structure, prevails, also explaining the large work function reduction found for IPr-NHC that indicates a large charge transfer to the surface only consistent with a covalent-bond formation.

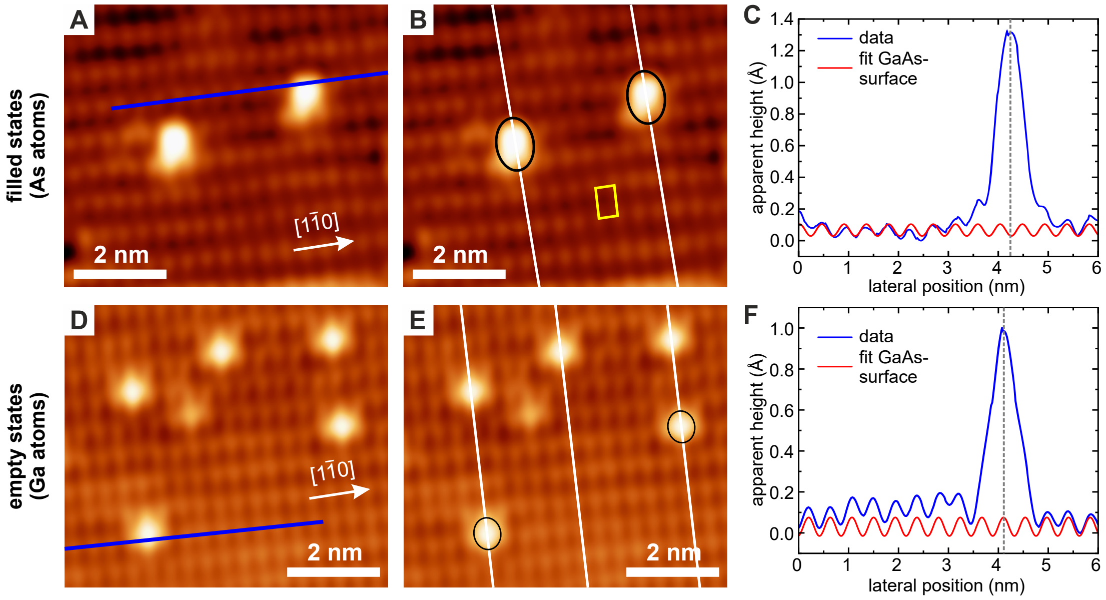


**Figure S13.** Atomically resolved (A,B) filled states ($V_T = -3.5$ V; $I_T = 20$ pA) and (D,E) empty states STM images ($V_T = +2.3$ V; $I_T = 20$ pA) of isolated IMe-NHC molecules on GaAs(110) and (C,F) corresponding height profiles (blue) along the lines indicated in (A) and (D). In (B) and (E), white lines are shown along the positions of Ga atoms appearing dark or bright in the filled and empty states image, respectively. The yellow box indicates the unit cell of the GaAs(110) surface. The red lines in (C,F) show sine fits of the surrounding GaAs surface highlighting the positions of the As and of the Ga atoms, respectively.

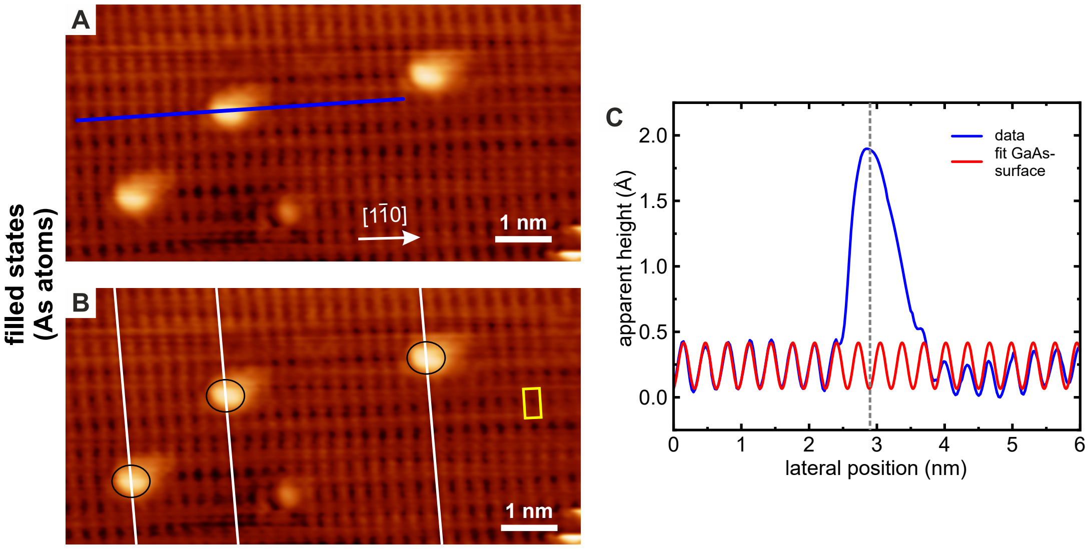


**Figure S14.** (A,B) Atomically resolved filled states STM image ($V_T = -2.5$ V; $I_T = 20$ pA) of isolated IMe-NHO molecules on GaAs(110) and (C) corresponding height profile (blue) along the line indicated in (A). In (B), white lines are shown along the positions of Ga atoms appearing dark in the filled states image. The yellow box indicates a unit cell of the GaAs(110) surface. The red line in (C) shows a sine fit of the surrounding GaAs surface.

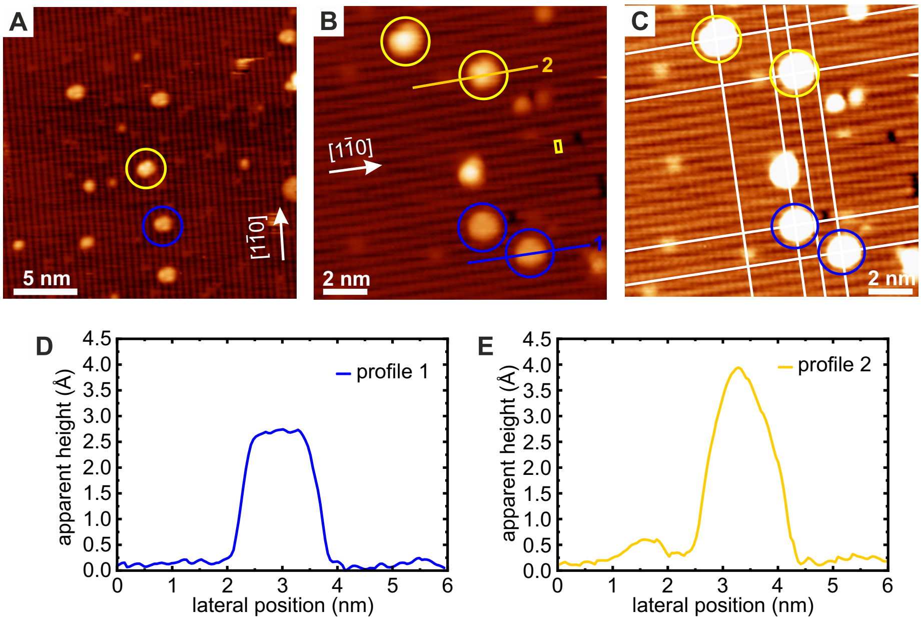


**Figure S15.** (A) Overview ($V_T = -2.8$ V; $I_T = 15$ pA) and (B,C) atomically resolved STM images ($V_T = -3.3$ V; $I_T = 10$ pA) of a low IPr-NHC coverage on GaAs(110) with (C) contrast enhanced at the GaAs surface to display the As atoms. The two different appearances are indicated by blue and yellow circles. (D,E) Height profiles along the lines indicated in (B).

# 9. Detailed Monolayer Structure

As discussed in the main text, BIMe-NHC shows the most well-ordered monolayer forming a long-range $(5 \times 1)$ superstructure, as demonstrated by LEED and STM. Figure S16 presents an accurate measurement of the separations between the BIMe-NHC chains by fitting each molecule by a Gaussian peak in the height profile. The obtained peak positions are given in Figure S16(B) and Table S3 together with their separations in both nm and units of the lattice constant $a_{[1\bar{1}0]}$ in that direction. It can be clearly seen that the large separation is always smaller than $3a_{[1\bar{1}0]}$ and the short one is always larger than $2a_{[1\bar{1}0]}$ showing the outward bending of the molecular chain pair with closer separation distance.

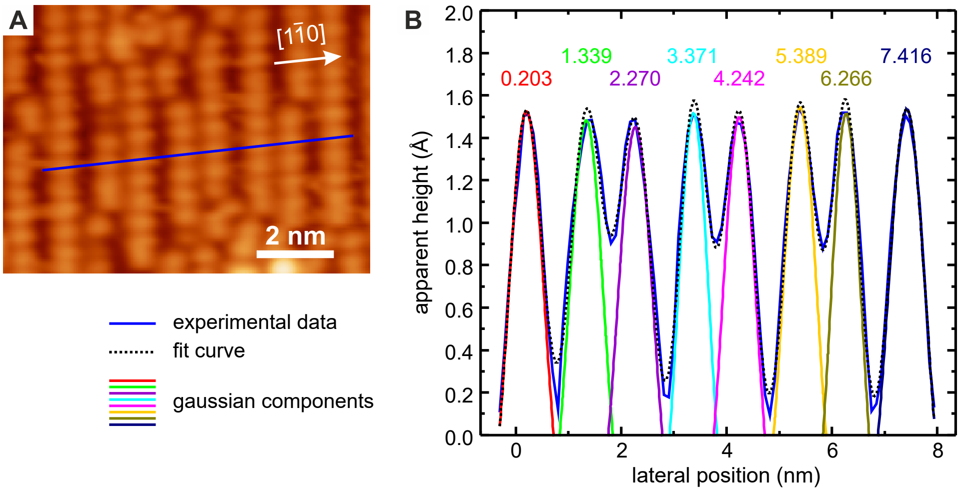


**Figure S16.** Determination of the precise distances in the BIMe-NHC monolayer. (A) STM image ($V_T = -3.6$ V; $I_T = 15$ pA) and (B) height profile along the blue line in (A), as already shown in Figure 5. In (B), the peaks were fitted with Gaussian curves and the obtained peak positions are marked.

**Table S3.** Determination of chain separation for the height profile shown in Figure S16.

| Peak | Position (nm) | Distance to previous peak (nm) | Distance to previous peak ($a_{[1\bar{1}0]}$) |
|---|---|---|---|
| 1 | 0.20 | - | - |
| 2 | 1.34 | 1.14 | 2.84 |
| 3 | 2.27 | 0.93 | 2.32 |
| 4 | 3.37 | 1.10 | 2.75 |
| 5 | 4.24 | 0.87 | 2.18 |
| 6 | 5.39 | 1.15 | 2.86 |
| 7 | 6.27 | 0.88 | 2.19 |
| 8 | 7.42 | 1.15 | 2.87 |

# 10. Coverage Determination

The molecular coverage in the monolayers is determined from both STM and XPS data. For STM, the number of molecules is counted in overview images, which were precisely calibrated at the clean GaAs(110) surface. For XPS, the coverages are determined from the N/Ga, N/As, C/Ga, and C/As ratios. The intensities are corrected by the respective photoionization cross sections[31] and the inelastic mean free path was estimated on the basis of the TPP-2M equation by Tanuma et al.[32] The obtained values are given in Table S4.

**Table S4.** Molecular coverages in the NHC and NHO monolayers as determined from STM and XPS data given in molecules per GaAs(110) unit cell. Coverage from XPS represents the average between of the values obtained from the different ratios.

| Molecule | Coverage from STM | Coverage from XPS |
|---|---|---|
| BIMe-NHC | $0.37 \pm 0.03$ | $0.37 \pm 0.04$ |
| IMe-NHC | $0.35 \pm 0.03$ | $0.33 \pm 0.03$ |
| IMe-NHO | $0.35 \pm 0.03$ | $0.32 \pm 0.04$ |
| IPr-NHC | $0.12 \pm 0.03$ | $0.15 \pm 0.04$ |

It should be noted that for all systems studied here, the stable molecular coverage saturates at a full monolayer and no further molecular layers form on top of the monolayers. To ensure a full coverage of the samples in the XPS measurements, it was always checked that the intensity of the C 1$s$ signal is in saturation.

## 11. Optical Properties Calculations

It is natural to interpret the positive RAS signal at 3.8 eV initially as deriving from optical transitions within the NHC molecules, especially since the heterocyclic rings of the IMe-NHC and BIMe-NHC molecules in particular are aligned along the [1$\bar{1}$0] direction. To investigate this hypothesis we first performed GW and GW-BSE calculations of the gas phase BIMe-NHC molecule using the molGW code.[18] These post-DFT methods for computing quasiparticle energy levels and optical spectra including excitonic effects, respectively, yield a fairly accurate estimation of the HOMO-LUMO gap and the optical gap (lowest energy transition) and can thus be compared with experiment.[33]

In Figure S17(A) we report the DFT (PBE and B3LYP[34] functionals) and GW energy levels. While the DFT-PBE gap is only 3.4 eV, the true (GW) HOMO-LUMO gap is much larger, around 9.0 eV. Thanks to strong excitonic coupling within the molecule, the optical gap is reduced to 4.8 eV as predicted by GW-BSE (Figure S17(B)), although the lowest energy transition parallel to the heterocyclic plane (xx-component) is a little higher, at 5.3 eV. Clearly, these energies are far higher than the measured peak in the RAS at 3.8 eV, even accounting for estimated errors in the BSE peak position of ~0.3 eV, and rules out pure intramolecular excitations as being responsible for the induced RAS signal. Instead, as discussed in the main text, it can be related to transitions between the As dangling bond states and the molecular LUMO, also explaining the broad peak due to the width of the As-related surface state band.

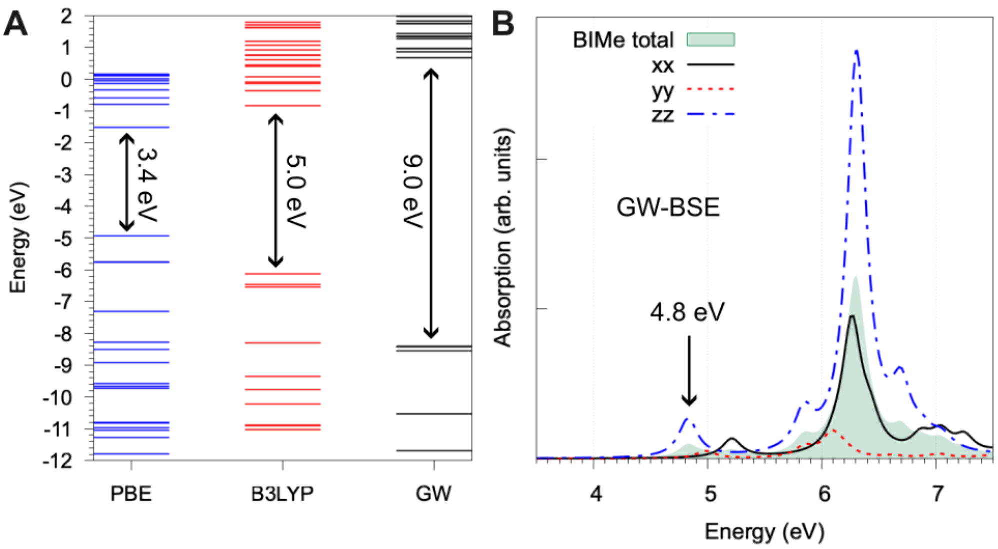


**Figure S17.** (A) Electronic energy levels of gas phase BIMe-NHC computed at different levels of theory. (B) Optical absorption spectra of BIMe-NHC. The average spectrum and its tensor components are shown.

## 12. Determination of the Work Function Change

Figure S18 shows the experimental data for the secondary electron (SE) onsets used to determine the change in work function. In all cases, spectra for the clean as well as for the monolayer covered GaAs(110) samples were measured. The energy axis shows the kinetic energy corrected by the applied bias of −9 V. The energy difference between the onsets gives the work function change and is determined by linear fits to the experimental data as shown by gray dashed lines. The results together with the change in band bending, as determined from the Ga 3*d* and As 3*d* core-level spectra shown in Figure S21, are summarized in Table S5.

As discussed in the main text, the largest reduction of −2.31 eV is found for IMe-NHO and related to two effects: first, the SE onset for the clean surface, which slightly varies from cleavage to cleavage, is with 4.61 eV higher in energy as for the other molecules showing values around 4.4 eV. However, this can only explain part of the large work function reduction. The second effect playing a role here is the orientation of the internal molecular dipole moment. As the IMe-NHO adsorption geometry is closest to vertical for the molecules with methyl sidegroups, it may be expected that also the vertical component of the internal molecular dipole moment is largest for this molecule, leading also to the lowest final value for the SE onset for IMe-NHO and thus the largest work function reduction.

Additionally, the band bending changes when the molecules adsorb, as observed by energy shifts in the Ga 3*d* and As 3*d* core-level spectra shown in Figure S21. The change in band bending is determined as the average from the spectra measured under NE and 70° and is also given in Table S5. Taking this into account, also the change in the electron affinity can be calculated as the sum of the work function change and the band bending change, as also given in Table S5.

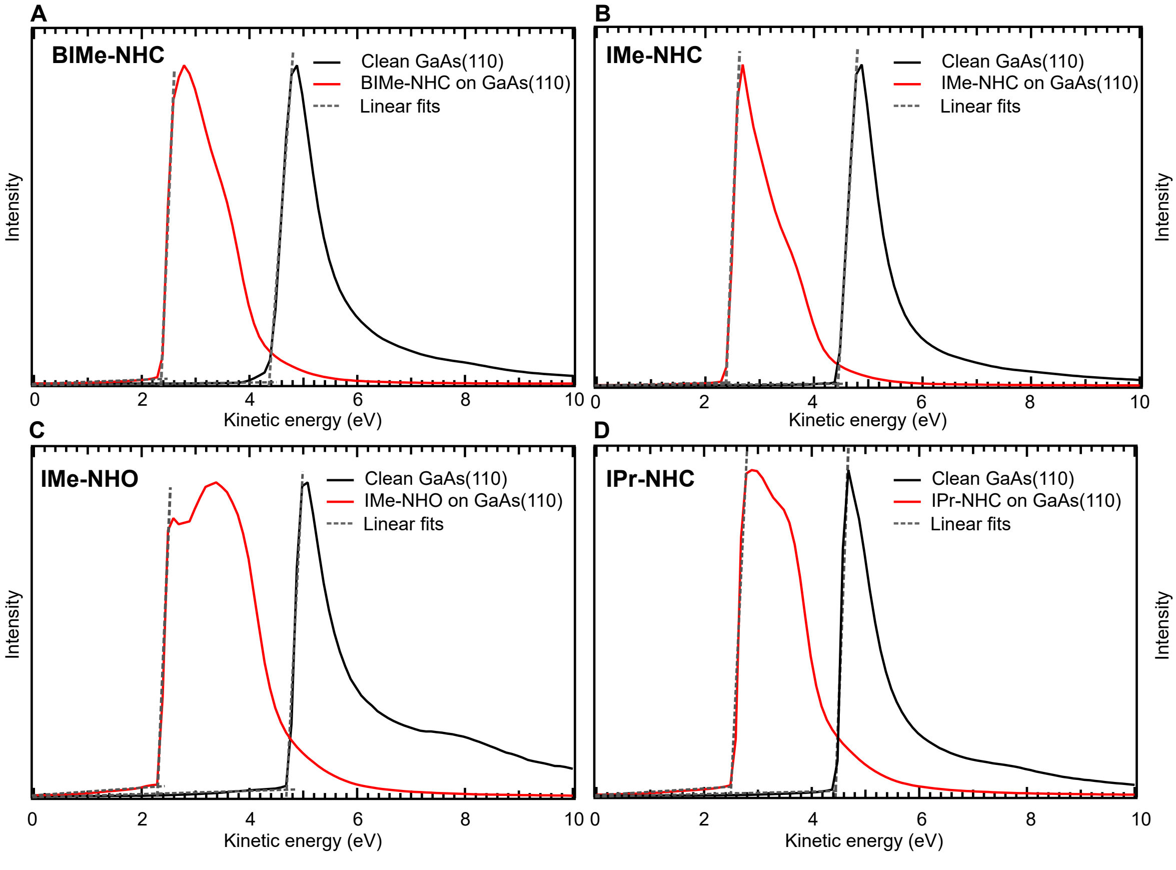


**Figure S18.** XPS measurements of the secondary electron onsets for the clean GaAs(110) surface (black) and monolayers of (A) BIMe-NHC, (B) IMe-NHC, (C) IMe-NHO, and (D) IPr-NHC (red). The onsets are determined from linear fits to the data as shown by the dashed gray lines. For better comparability, the spectra were normalized to equal height.

**Table S5.** Measured kinetic energies of the secondary electron (SE) onsets for the GaAs surfaces prior and after formation of the NHC and NHO monolayers, change in band bending, resulting work function changes $\Delta W$, and electron affinity changes $\Delta E_A$.

| Molecule | BIMe-NHC | IMe-NHC | IMe-NHO | IPr-NHC | Uncertainty |
|---|---|---|---|---|---|
| SE onset clean GaAs (eV) | 4.36 | 4.44 | 4.61 | 4.41 | ±0.05 |
| SE onset monolayer (eV) | 2.36 | 2.38 | 2.30 | 2.50 | ±0.05 |
| Band bending change (eV) | −0.14 | −0.13 | −0.26 | −0.12 | ±0.05 |
| $\Delta W$ (eV) | −2.00 | −2.06 | −2.31 | −1.91 | ±0.10 |
| $\Delta E_A$ (eV) | −2.14 | −2.19 | −2.57 | −2.03 | ±0.15 |

# 13. Overview XPS Spectra

In Figure S19, overview XPS spectra of the clean surfaces and the monolayers of all investigated molecules are presented. The data prove the successful deposition of the molecules by the exclusive appearance of weak signals at the positions of the C 1*s* and N 1*s* photoelectron lines (see indications). All other peaks in the spectra can be assigned to photoelectron or Auger lines from the GaAs substrate, as indicated, while the absence of additional lines, e.g. of an O 1*s* signal, demonstrates the cleanliness of the preparation without any contamination.

# 14. C 1*s* and N 1*s* Core Levels

For further XPS characterization, C 1*s* and N 1*s* core-level spectra were measured in detail and fitted for all molecules. The results are presented in Figure S20. For BIMe-NHC, IMe-NHC, and IMe-NHO, the C 1*s* signals are decomposed into several components representing the chemically different C atoms. All spectra can be satisfactorily fit using these components with intensity ratios according to the occurrence of the C atoms in the respective molecule. Due to the much more complex structure of the IPr-NHC molecule, such a simple decomposition is not possible as discussed e.g. in our previous work, in which the spectrum was decomposed based on theoretical predictions.[7] As such an analysis is beyond the scope of the present work, the IPr-NHO spectrum is fitted with the least number of components needed to obtain a satisfactory fit, i.e. with four components.

The N 1*s* spectra can all be fitted with a single component. For the case of the molecules with methyl sidegroups this is expected, as all three molecules contain two chemically identical N atoms, and as all adsorption geometries are symmetric with respect to the N atoms. In the case of IPr-NHC, at least very similar N 1*s* binding energies are expected, since the two N atoms are also chemically identical and placed in similar positions relative to the GaAs substrate.

# 15. Ga 3*d* and As 3*d* Core Levels

In Figure S21 spectra for the Ga 3*d* and the As 3*d* core levels are shown, each measured for the respective clean and molecule covered GaAs(110) surface. From the shift between the peaks, the values for the change in band bending given in Table S5 were determined.

Surface photovoltage effects in photoemission experiments can be ruled out here, since there were no such effects reported at room temperature for photon intensities typical for the present XPS experiments.[35,36] Moreover, in the case of surface photovoltage effects, spectral shifts should be detected between the NE and 70° spectra in Figure S21 due to the different photon injection geometries resulting in different X-ray intensities. No such shifts are observed here.

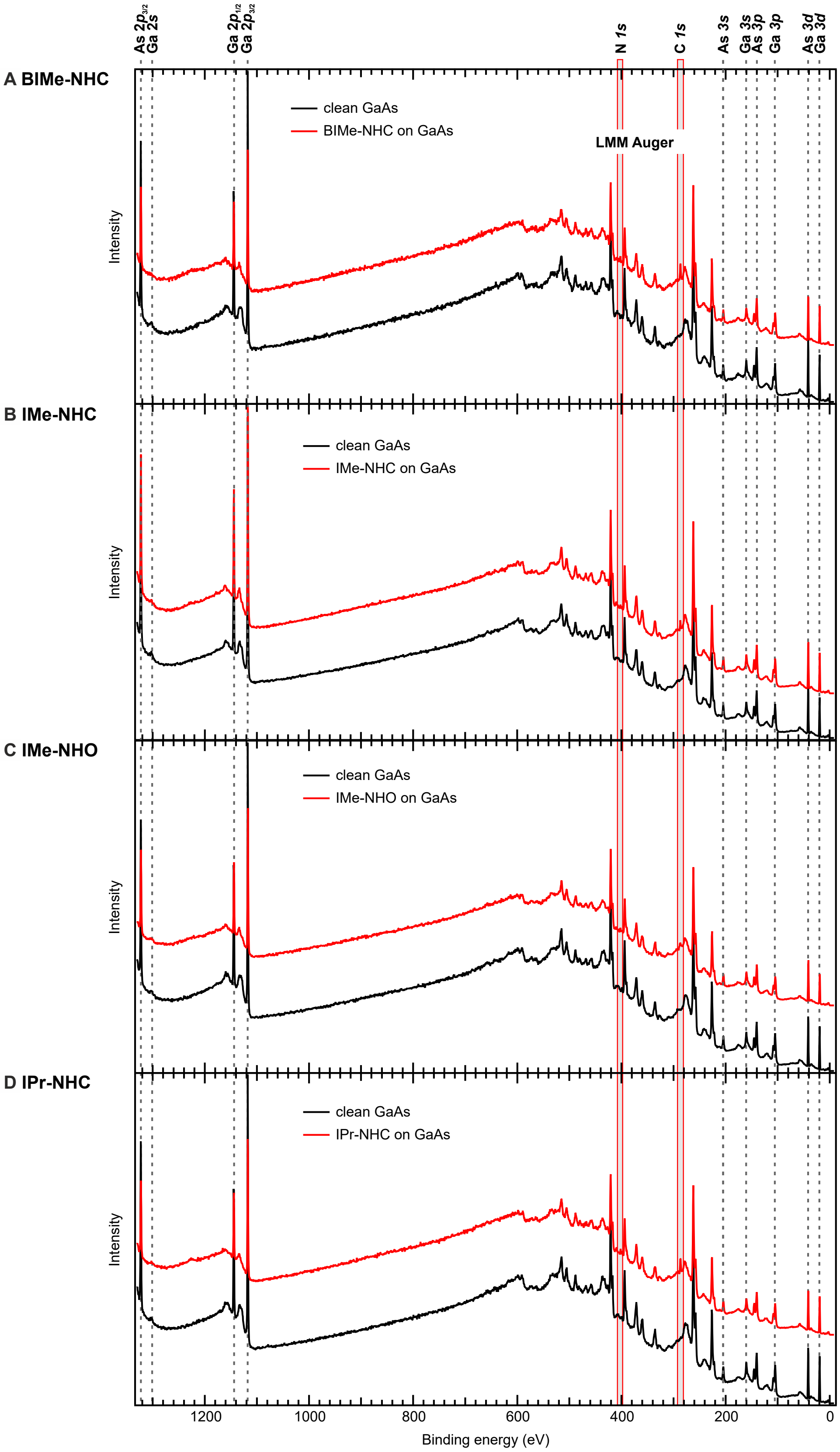


**Figure S19.** Overview XPS spectra for the clean GaAs(110) surface (black) and monolayers of (A) BIMe-NHC, (B) IMe-NHC, (C) IMe-NHO, and (D) IPr-NHC on GaAs(110) (red).

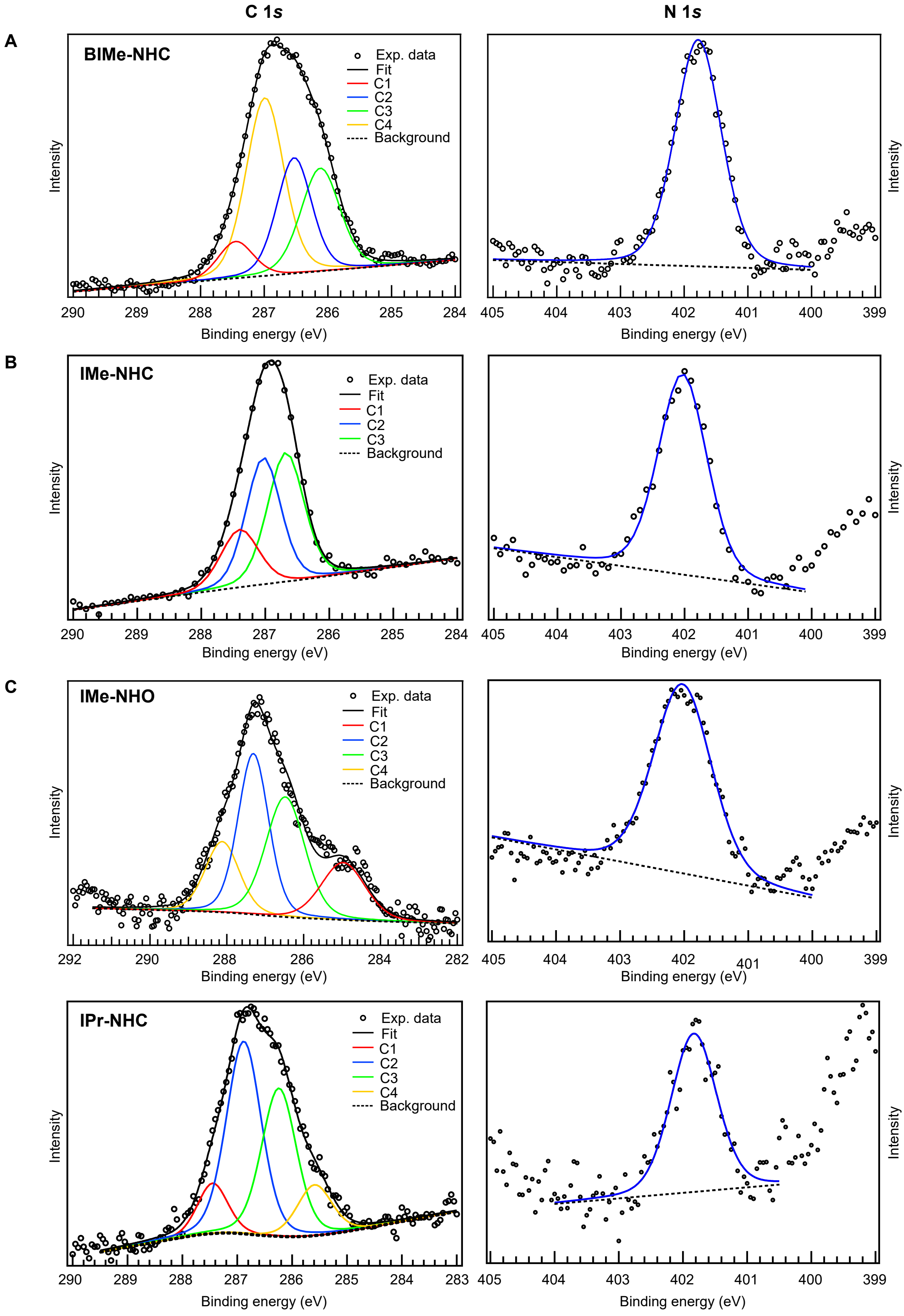


**Figure S20.** XPS results for the C $1s$ and N $1s$ core-levels for monolayers of (A) BIMe-NHC, (B) IMe-NHC, (C) IMe-NHO, and (D) IPr-NHC on GaAs(110).

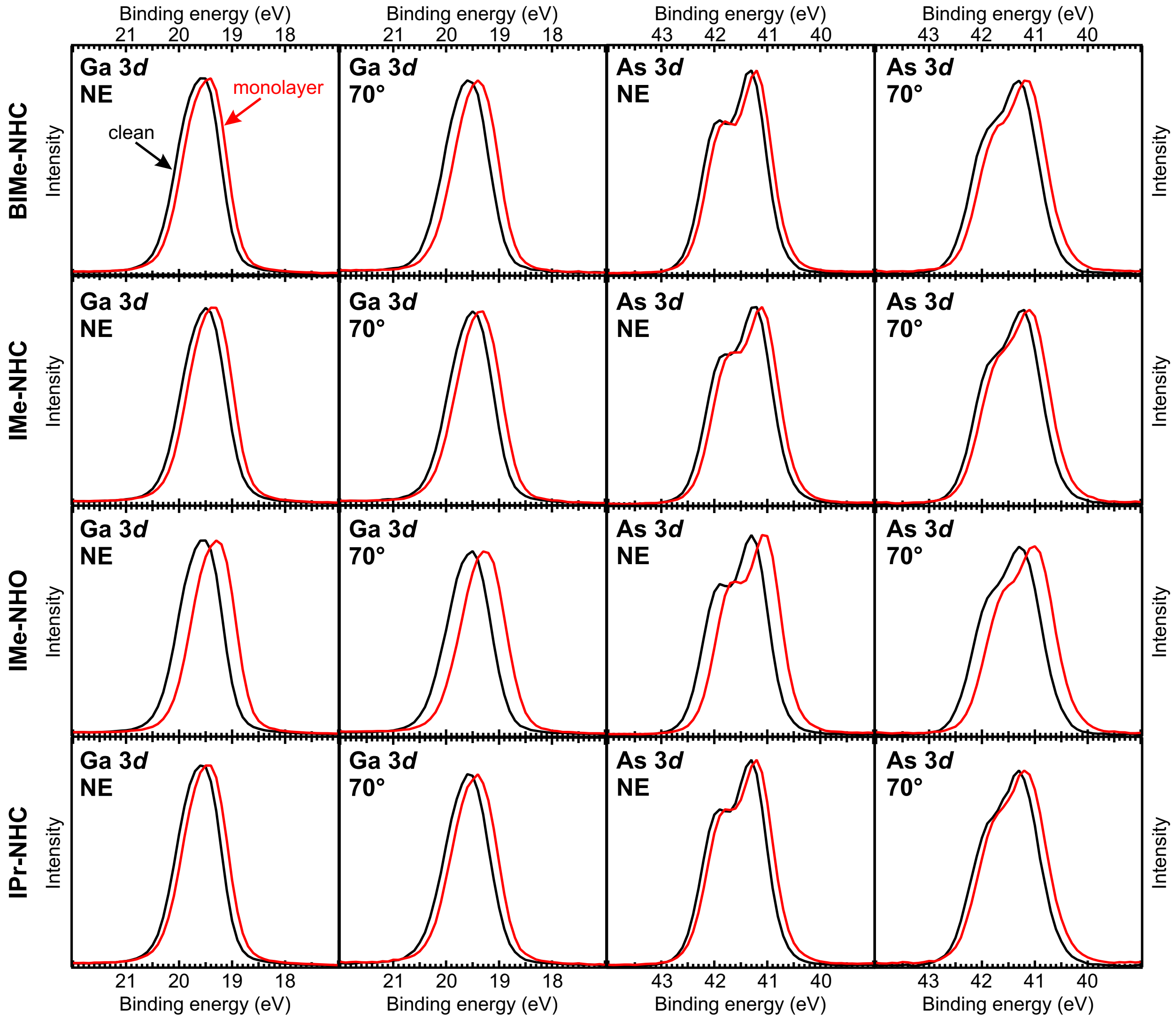


**Figure S21.** XPS results for the Ga $3d$ and the As $3d$ core-levels for clean (black) and monolayer covered GaAs(110) (red) measured under normal emission (NE) and under an angle of 70° with respect to the surface normal. For better comparability, the spectra were normalized to equal height.

## References


[1] D. E. Aspnes, A. A. Studna, *Phys. Rev. Lett.* **1985**, *54*, 1956.
[2] H. Lüth, *Solid Surfaces, Interfaces and Thin Films*, Springer, Cham, 6 edition **2014**.
[3] J. W. Kim, A. Kim, *Curr. Appl. Phys.* **2021**, *31*, 52.
[4] T. Vaara, J. Vaari, J. Lahtinen, *Surf. Sci.* **1998**, *395*, 88.
[5] P. Finetti, M. Scantlebury, R. McGrath, F. Borgatti, M. Sambi, L. Zaratin, G. Granozzi, *Surf. Sci.* **2000**, *461*, 240.
[6] M. Prietsch, M. Domke, C. Laubschat, T. Mandel, C. Xue, G. Kaindl, *Z. Phys. B Condens. Matter* **1989**, *74*, 21.
[7] M. Franz, S. Chandola, M. Koy, R. Zielinski, H. Aldahhak, M. Das, M. Freitag, U. Gerstmann, D. Liebig, A. K. Hoffmann, M. Rosin, W. G. Schmidt, C. Hogan, F. Glorius, N. Esser, M. Dähne, *Nat. Chem.* **2021**, *13*, 828–835.
[8] R. Zielinski, M. Das, C. Kosbab, M. T. Nehring, M. Dähne, N. Esser, M. Franz, F. Glorius, *J. Mater. Chem. C* **2023**, *11*, 7377.
[9] M. Das, C. Hogan, R. Zielinski, M. Kubicki, M. Koy, C. Kosbab, S. Brozzesi, A. Das, M. T. Nehring, V. Balfanz, J. Brühne, M. Dähne, M. Franz, N. Esser, F. Glorius, *Angew. Chem. Int. Ed.* **2023**, *62*, e202314663.
[10] F. Landwehr, M. Das, S. Tosoni, J. J. Navarro, A. Das, M. Koy, M. Heyde, G. Pacchioni, F. Glorius, B. R. Cuenya, *Adv. Mater. Interfaces* **2024**, *11*, 2400378.
[11] G. Wang, A. Rühling, S. Amirjalayer, M. Knor, J. B. Ernst, C. Richter, H.-J. Gao, A. Timmer, H.-Y. Gao, N. L. Doltsinis, F. Glorius, H. Fuchs, *Nat. Chem.* **2017**, *9*, 152.
[12] P. Giannozzi, O. Andreussi, T. Brumme, O. Bunau, M. Buongiorno Nardelli, M. Calandra, R. Car, C. Cavazzoni, D. Ceresoli, M. Cococcioni, N. Colonna, I. Carnimeo, A. Dal Corso, S. de Gironcoli, P. Delugas, R. A. DiStasio, A. Ferretti, A. Floris, G. Fratesi, G. Fugallo, R. Gebauer, U. Gerstmann, F. Giustino, T. Gorni, J. Jia, M. Kawamura, H.-Y. Ko, A. Kokalj, E. Küçükbenli, M. Lazzeri, M. Marsili, N. Marzari, F. Mauri, N. L. Nguyen, H.-V. Nguyen, A. Otero-de-la Roza, L. Paulatto, S. Poncé, D. Rocca, R. Sabatini, B. Santra, M. Schlipf, A. P. Seitsonen, A. Smogunov, I. Timrov, T. Thonhauser, P. Umari, N. Vast, X. Wu, S. Baroni, *J. Phys. Condens. Matter* **2017**, *29*, 465901.
[13] S. Grimme, J. Antony, S. Ehrlich, H. Krieg, *J. Chem. Phys.* **2010**, *132*.
[14] A. Ravikumar, A. Baby, H. Lin, G. P. Brivio, G. Fratesi, *Sci. Rep.* **2016**, *6*, 24603.
[15] K. Momma, F. Izumi, *J. Appl. Crystallogr.* **2011**, *44*, 1272.
[16] C. Fonseca Guerra, J.-W. Handgraaf, E. J. Baerends, F. M. Bickelhaupt, *J. Comput. Chem.* **2004**, *25*, 189.
[17] A. O. de-la Roza, E. R. Johnson, V. Luaña, *Comput. Phys. Commun.* **2014**, *185*, 1007.
[18] F. Bruneval, T. Rangel, S. M. Hamed, M. Shao, C. Yang, J. B. Neaton, *Comput. Phys. Commun.* **2016**, *208*, 149.
[19] R. A. Kendall, J. Dunning, Thom H., R. J. Harrison, *J. Chem. Phys.* **1992**, *96*, 6796.
[20] B. R. Van Ausdall, J. L. Glass, K. M. Wiggins, A. M. Aarif, J. Louie, *J. Org. Chem.* **2009**, *74*, 7935.
[21] C. M. Crudden, J. H. Horton, M. R. Narouz, Z. Li, C. A. Smith, K. Munro, C. J. Baddeley, C. R. Larrea, B. Drevniok, B. Thanabalasingam, A. B. McLean, O. V. Zenkina, I. I. Ebralidze, Z. She, H.-B. Kraatz, N. J. Mosey, L. N. Saunders, A. Yagi, *Nat. Commun.* **2016**, *7*, 12654.
[22] A. R. Lubinsky, C. B. Duke, B. W. Lee, P. Mark, *Phys. Rev. Lett.* **1976**, *36*, 1058.
[23] J. R. Chelikowsky, S. G. Louie, M. L. Cohen, *Phys. Rev. B* **1976**, *14*, 4724.
[24] D. J. Chadi, *Phys. Rev. Lett.* **1978**, *41*, 1062.
[25] D. J. Chadi, *Phys. Rev. B* **1979**, *19*, 2074.
[26] J. L. A. Alves, J. Hebenstreit, M. Scheffler, *Phys. Rev. B* **1991**, *44*, 6188.
[27] O. Pulci, G. Onida, R. Del Sole, A. J. Shkrebtii, *Phys. Rev. B* **1998**, *58*, 1922.
[28] R. M. Feenstra, J. A. Stroscio, J. Tersoff, A. P. Fein, *Phys. Rev. Lett.* **1987**, *58*, 1192.
[29] D. E. Eastman, T. C. Chiang, P. Heimann, F. J. Himpsel, *Phys. Rev. Lett.* **1980**, *45*, 656.
[30] L. Sorba, M. Pedio, S. Nannarone, S. Chang, A. Raisanen, A. Wall, P. Philip, A. Franciosi, *Phys. Rev. B* **1990**, *41*, 1100.
[31] J. Scofield, *J. Electron Spectrosc. Relat. Phenom.* **1976**, *8*, 129.
[32] S. Tanuma, C. J. Powell, D. R. Penn, *Surf. Interface Anal.* **1994**, *21*, 165.
[33] F. Bruneval, S. M. Hamed, J. B. Neaton, *J. Chem. Phys.* **2015**, *142*, 244101.
[34] A. D. Becke, *J. Chem. Phys.* **1993**, *98*, 5648.
[35] M. H. Hecht, *Phys. Rev. B* **1990**, *41*, 7918.
[36] A. Bauer, M. Prietsch, S. Molodtsov, C. Laubschat, G. Kaindl, *J. Vac. Sci. Technol. B* **1991**, *9*, 2108.
